# NQO1 regulates cell cycle progression at the G2/M phase

**DOI:** 10.7150/thno.77444

**Published:** 2023-01-10

**Authors:** Eun-Taex Oh, Ha Gyeong Kim, Chul Hoon Kim, Jeonghun Lee, Chulhee Kim, Jae-Seon Lee, Yunmi Cho, Heon Joo Park

**Affiliations:** 1 Department of Biomedical Sciences, College of Medicine, Inha University, Incheon 22212, Republic of Korea; 2 Program in Biomedical Science & Engineering, Inha University, Incheon 22212, Republic of Korea; 3 Department of Pharmacology, Yonsei University College of Medicine, Seoul 03722, Republic of Korea; 4 Department of Polymer Science & Engineering, Inha University, Incheon 22212, Republic of Korea; 5 Department of Molecular Medicine, College of Medicine, Inha University, Incheon 22212, Republic of Korea; 6 Research Center for Controlling Intracellular Communication, College of Medicine, Inha University, Incheon 22212, Republic of Korea; 7 Department of Microbiology, College of Medicine, Inha University, Incheon 22212, Republic of Korea

**Keywords:** NQO1, cancer cell, cell cycle, c-Fos, CKS1

## Abstract

**Rationale:** Overexpression of NAD(P)H:quinone oxidoreductase 1 (NQO1) is associated with tumor cell proliferation and growth in several human cancer types. However, the molecular mechanisms underlying the activity of NQO1 in cell cycle progression are currently unclear. Here, we report a novel function of NQO1 in modulation of the cell cycle regulator, cyclin-dependent kinase subunit-1 (CKS1), at the G2/M phase through effects on the stability of c‑Fos.

**Methods:** The roles of the NQO1/c-Fos/CKS1 signaling pathway in cell cycle progression were analyzed in cancer cells using synchronization of the cell cycle and flow cytometry. The mechanisms underlying NQO1/c-Fos/CKS1-mediated regulation of cell cycle progression in cancer cells were studied using siRNA approaches, overexpression systems, reporter assays, co-immunoprecipitation, pull-down assays, microarray analysis, and CDK1 kinase assays. In addition, publicly available data sets and immunohistochemistry were used to investigate the correlation between NQO1 expression levels and clinicopathological features in cancer patients.

**Results:** Our results suggest that NQO1 directly interacts with the unstructured DNA-binding domain of c-Fos, which has been implicated in cancer proliferation, differentiation, and development as well as patient survival, and inhibits its proteasome-mediated degradation, thereby inducing CKS1 expression and regulation of cell cycle progression at the G2/M phase. Notably, a NQO1 deficiency in human cancer cell lines led to suppression of c-Fos-mediated CKS1 expression and cell cycle progression. Consistent with this, high NQO1 expression was correlated with increased CKS1 and poor prognosis in cancer patients.

**Conclusions:** Collectively, our results support a novel regulatory role of NQO1 in the mechanism of cell cycle progression at the G2/M phase in cancer through effects on c‑Fos/CKS1 signaling.

## Introduction

NAD(P)H:quinone oxidoreductase 1 (NQO1) is a cytosolic reductase that plays an important role in cellular responses to oxidative stress 1. Numerous human cancers, including colorectal cancer 1, lung cancer 2, breast cancer 3, cholangiocarcinoma 4, ovarian cancer 5, uterine cervical cancer 4, pancreatic cancer 4, prostate cancer 5 and head-and-neck cancer 6, express 5- to 200-fold higher levels of NQO1 than their healthy tissue counterparts. NQO1 is intimately linked with multiple carcinogenic processes 7-10. In breast, colorectal, ovarian and cervical cancer types, elevated expression of NQO1 is closely associated with poor prognosis 3-5. While the implications of NQO1 expression in clinicopathological features and prognosis are clear, the underlying rationale for upregulation of NQO1 and its specific role in the development of solid tumors remain to be clarified. To our knowledge, very few studies to date have focused on the functions and mechanisms of action of NQO1 in cancer cell proliferation and growth.

NQO1 protects cells against various cytotoxic quinones and oxidative stress and catalyzes the reduction and detoxification of quinone substrates, thereby preventing cytotoxic effects of carcinogens 11-15. Considerable efforts have been made to develop bioreductive anticancer drugs, such as mitomycin C, E09, RH1, β-lapachone and 17AAG, that are activated specifically by NQO1 and thus preferentially kill cancer cells 16-24 by virtue of the unique ability of NQO1 to transfer two electrons using either NADH or NADPH as the reducing cofactor 25-27. Ionizing radiation (2-4 Gy) 27-30, cisplatin 30 and hyperthermia (41-42ºC) 31,32 are reported to increase NQO1 expression in various human and animal cancer cells and sensitize cells to β-lapachone, both *in vitro* and *in vivo*. Previous studies have disclosed protective effects of NQO1 on proteins that are independent of its enzymatic activity 33. For instance, NQO1 structurally binds the critical tumor-suppressor protein p53 and increases its stability by inhibiting proteasomal degradation 33. It has also been shown to regulate the stability of several other proteins, including p73, p33ING1b, C/EBPα, c-Fos, and HIF-1α 34-40. In view of these earlier findings, NQO1 is considered a multifunctional antioxidant enzyme and an exceptionally versatile cytoprotective molecule with a dual role in tumorigenic progression.

Previously, our group reported that NQO1 in cancer cells induces rapid degradation of Aurora-A during mitotic progression, specifically demonstrating that an NQO1 deficiency leads to aneuploidy during mitotic progression in irradiated cancer cells 41. In human cells, NQO1 is associated with mitotic spindles during mitotic progression 42 and has been further shown to regulate mitotic progression and response to mitotic stress through modulation of SIRT2 activity 43. However, the mechanisms underlying the activity of NQO1 in cancer cell proliferation, in particular, cell cycle progression, have yet to be clarified.

Cell cycle progression is a highly ordered process regulated by the oscillating expression of positive and negative factors 44. Coordination of the cell cycle is dependent on multiple interactions among cyclins, cyclin-dependent kinases (CDKs), and their inhibitors 44. One central regulatory protein with activity at G1-S and G2-M transition phases is cyclin-dependent kinase regulatory subunit 1 (CKS1), a member of the conserved CKS protein family 45. Two paralogs of CKS proteins, CKS1 and CKS2, have been demonstrated to be essential for CDK function and division in human cells 46-48. CKS1, encoded by the *CKS1B* gene on human chromosome Iq21, has a molecular weight of 9 kDa and is highly functionally conserved 45. CKS1 was initially identified in fission yeast 47, where its loss of function was shown to result in mitotic defects 44. In mammalian cells, CKS proteins induce ubiquitylation and degradation of cyclin A complexed with Cdc20 at pre-anaphase, an action that is required for mitotic progression 44. In another earlier study, CKS1-depleted cells not only exhibited slower G1 phase progression but also accumulated at the G2/M phase owing to blockage of mitotic entry induced by the resultant decrease in CDK1 expression 49. CKS1 has additionally been reported to regulate S phase entry 44. CKS1 protein is commonly upregulated in association with the pathogenesis of multiple human cancers, including hepatocellular carcinoma, colon cancer, lung cancer, oral squamous cell carcinoma, breast cancer and retinoblastoma, and is significantly associated with cancer cell growth, invasion, metastasis, and drug resistance 45. Although both NQO1 and CKS1 are overexpressed in cancer, the potential interactions between the two proteins and their effects on the cell cycle have yet to be established.

Experiments from the current study support a novel role of NQO1 in the regulation of cell cycle progression at the G2/M phase in cancer cells. Taken together, our data suggest that NQO1 directly interacts with the DNA-binding domain (DBD) of c-Fos and inhibits its proteasome-mediated degradation, thereby increasing its stability. This NQO1-mediated increase in protein stability of c-Fos further promotes expression of CKS1 in cancer cells, resulting in increased cell proliferation and radioresistance. High-level expression of NQO1 is consistently correlated with elevated expression of CKS1 and poor survival in cancer patients.

## Results

### NQO1 regulates cancer cell proliferation

**Figure 1:**
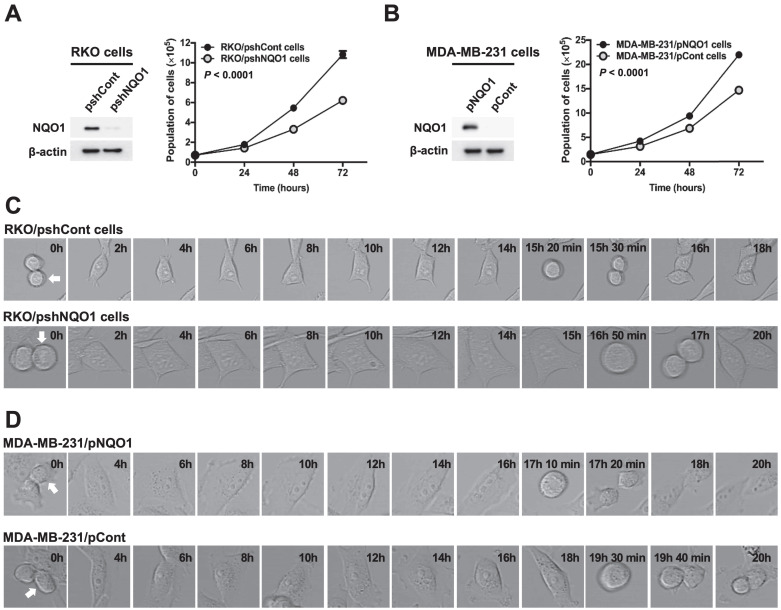
** NQO1 regulates cancer cell proliferation. (A-B)** RKO/pshCont and RKO/pshNQO1 cells **(A)** and MDA-MB-231/pNQO1 and MDA-MB-231/pCont cells **(B)** were seeded in T25 flasks and the cell numbers determined daily. All error bars represent mean ± SD. RKO/pshCont cells proliferated more than RKO/pshNQO1 cells (*P* < 0.0001) **(A)**. MDA-MB-231/pNQO1 cells proliferated more than MDA-MB-231/pCont cells (*P* < 0.0001) **(B)**. **(C-D)** RKO/pshCont and RKO/pshNQO1 cells **(C)** and MDA-MB-231/pNQO1 and MDA-MB-231/pCont cells **(D)** were seeded in 8-well chamber slides and cell cycle progression determined via confocal microscopy as indicated. White arrows signify the cells monitored for analysis of cell cycle progression.

Gain-of-function and loss-of-function experiments were conducted to establish the effects of NQO1 on cancer cell proliferation. To this end, RKO human colorectal cancer cells were transfected with small inhibitory (interfering) RNA (shRNA) targeting NQO1 (RKO/pshNQO1) or control scrambled shRNA (RKO/pshCont). As shown in Figure 1A, depletion of NQO1 (RKO/pshNQO1 cells) resulted in slower proliferation of cancer cells relative to that in the control group. In gain-of-function experiments, NQO1 expression in NQO1-deficient MDA-MB-231 human breast cancer cells (MDA-MB-231/pNQO1 cells) induced a dramatic increase in cancer cell proliferation compared with parental NQO1-deficient MDA-MB-231/pCont cells (Figure 1B). To further examine the potential involvement of NQO1 in the regulation of cell cycle progression, we analyzed the cell cycle dynamics of cancer cells at the single-cell level with the aid of time-lapse confocal microscopy. In RKO/pshCont cells, 15 h 30 min were required from one cell division to the next, an interval that was increased to 17 h in NQO1-depleted RKO/pshNQO1 cells (Figure 1C and Figure S1A). To confirm these results, we additionally performed NQO1 gain-of-function experiments in MDA-MB-231 human breast cancer cells. Overall, 19 h 40 min were required from one cell division to the next in NQO1-deficient MDA-MB-231/pCont cells; this was decreased to 17 h 20 min in NQO1-overexpressing MDA-MB-231/pNQO1 cells (Figure 1D and Figure S1B). These data clearly implicate NQO1 in the regulation of cell cycle progression.

### NQO1 regulates cell cycle progression at the G2/M phase in cancer cells

**Figure 2:**
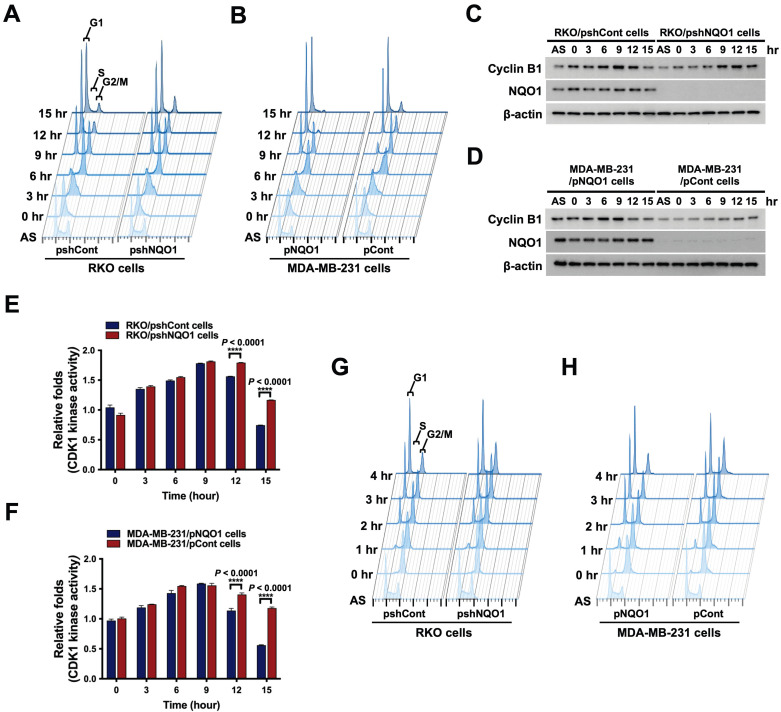
** NQO1 regulates cell cycle progression at the G2/M phase in cancer cells. (A-B)** RKO/pshCont and RKO/pshNQO1 cells **(A)** and MDA-MB-231/pNQO1 and MDA-MB-231/pCont cells **(B)** were synchronized at the G1/S boundary via double-thymidine blocking, subsequently released from synchronization, and analyzed for DNA content using flow cytometry. Experiments were conducted in triplicate. Data shown are representative of a typical experiment. AS indicates asynchronization. **(C-D)** RKO/pshCont and RKO/pshNQO1 cells **(C)** and MDA-MB-231/pNQO1 and MDA-MB-231/pCont cells **(D)** were synchronized at the G1/S boundary via double-thymidine blocking and released from synchronization. After incubation for the indicated times, cell lysates were subjected to immunoblot analysis using anti-cyclin B1, anti-NQO1, and anti-β-actin antibodies. AS indicates asynchronization. **(E-F)** RKO/pshCont and RKO/pshNQO1 cells **(E)** and MDA-MB-231/pNQO1 and MDA-MB-231/pCont cells **(F)** were synchronized at the G1/S boundary via double-thymidine blocking, released from synchronization and harvested at the indicated times. CDK1 kinase activities were analyzed using the CDK1 kinase assay kit. All data are presented as mean ± SEM. **** *P* < 0.0001 with ANOVA. **(G-H)** RKO/pshCont and RKO/pshNQO1 cells **(G)** and MDA-MB-231/pNQO1 and MDA-MB-231/pCont cells **(H)** were synchronized at the G2/M boundary via nocodazole blocking, released from synchronization and analyzed for DNA content using flow cytometry. Experiments were conducted in triplicate. Data are representative of a typical experiment. As indicates asynchronization.

To further ascertain the precise role of NQO1 in cancer cell cycle progression, we conducted a cell cycle analysis using cancer cells that were synchronized at the G1/S phase boundary via double-thymidine blocking and subsequently released to allow progression through the cell cycle. Cells were harvested at different time-points after release, as indicated, and analyzed by flow cytometry with propidium iodide (Figure 2A,B). The data showed a delay in cell cycle progression during the G2/M phase in NQO1-deficient RKO/pshNQO1 and MDA-MB-231/pCont cells (Figure 2A,B and Figure S2A,B). To validate the potential regulatory function of NQO1 in cell cycle progression at the G2/M phase in cancer cells, we determined the protein levels of associated cyclin B1 and CDK1. Cancer cells were again synchronized at the G1/S phase boundary via double-thymidine block, released, and harvested at different time points as indicated (Figure 2A,B and Figure S2A,B). Immunoblot analyses revealed increased expression of cyclin B1 at S and G2/M phases in NQO1-expressing RKO/pshCont and MDA-MB-231/pNQO1 cells, concomitant with a significant decrease in cyclin B1 levels at the G1 phase (Figure 2C,D). In contrast, expression of cyclin B1 was delayed in NQO1-deficient RKO/pshNQO1 and MDA-MB-231/pCont cells and its protein levels gradually decreased with cell cycle progression (Figure 2C,D).

Next, we focused on the potential regulatory effect of NQO1 on CDK1 in cancer cells. As shown in Figure S3A and B, we found that CDK1 expression is not dependent on NQO1 expression status. In view of these results, we hypothesized that NQO1 exerts regulatory effects on cell cycle progression at the G2/M phase in cancer cells. To test this, we investigated CDK1 activity in relation to expression of NQO1 in cancer cells. Consistent with flow cytometry and immunoblot data, after increasing at S phase, CDK1 kinase activity did not rapidly decrease after release of double-thymidine block in NQO1-deficient RKO/pshNQO1 and MDA-MB-231/pCont cells (Figure 2E,F). Figure 2E and F also reveal a difference in the degree of decrease in CDK1 activity between RKO/pshCont cells and MDA-MB-231/pNQO1 cells at 12 and 15 h, with CDK1 activity in MDA-MB-231/pNQO1 cells rapidly decreasing compared to that in RKO/pshCont cells. These results are consistent with flow cytometry results obtained for RKO/pshCont and MDA-MB-231/pNQO1 cells in the G2/M phase (Figure 2A,B). These results appear to reflect differences in proliferation rate between the two cell types resulting from the rapid acceleration of cell cycle progression induced by the overexpression of NQO1 in NQO1-deficient MDA-MB-231 cells. To confirm NQO1-mediated effects on cell cycle progression at the G2/M phase, we synchronized cancer cells at the G2/M phase boundary by blocking with nocodazole, then released and harvested cells at different time-points as indicated, followed by a flow cytometry analysis of cell cycle distribution. An NQO1 deficiency led to a delayed decrease in a large proportion of cells at the G2/M phase (RKO/pshNQO1 and MDA-MB-231/pCont cells), whereas the number of NQO1-expressing RKO/pshCont and MDA-MB-231/pNQO1 cells at the G2/M phase was rapidly decreased following nocodazole release (Figure 2G,H and Figure S4A,B). Collectively, these results clearly support effects of NQO1 on cell cycle progression at the G2/M phase in cancer cells.

### NQO1 regulates CKS1-mediated cell cycle progression at the G2/M phase in cancer cells

**Figure 3:**
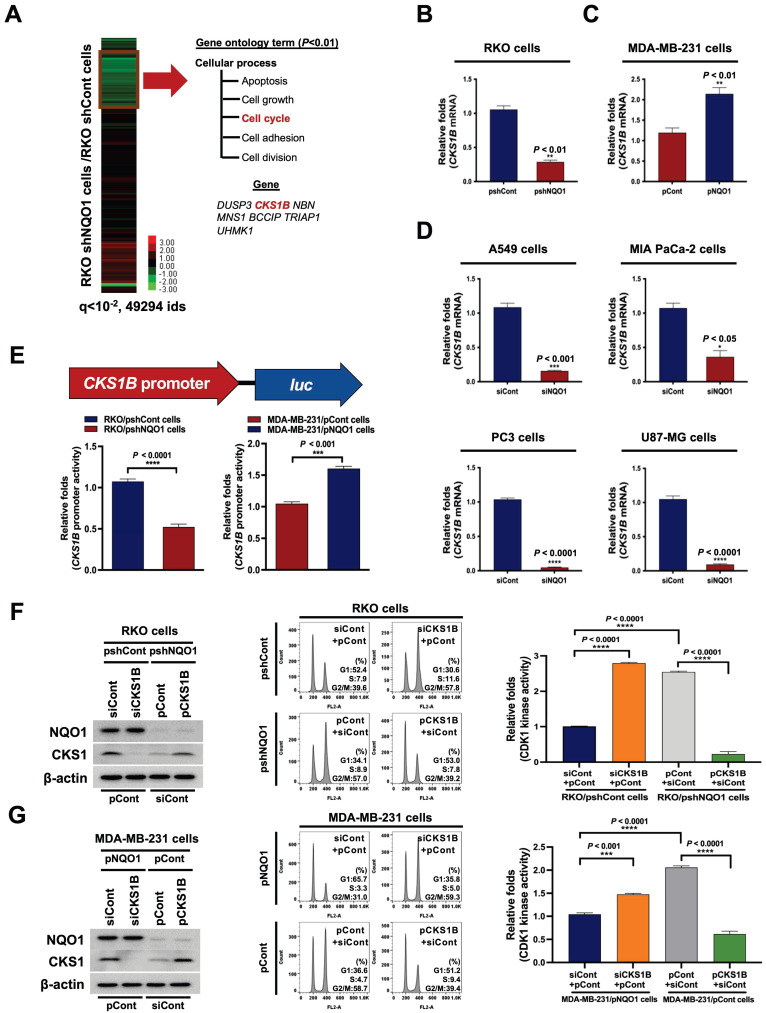
** NQO1 regulates CKS1-mediated cell cycle progression at the G2/M phase in cancer cells. (A)** Heatmap representation of microarray data on gene levels in RKO/pshCont and RKO/pshNQO1 cells. **(B-C)** Relative mRNA levels of *CKS1B* in RKO/pshCont and RKO/pshNQO1 cells **(B)** and MDA-MB-231/pNQO1 and MDA-MB-231/pCont cells **(C)**. *CKS1B* expression was examined via qPCR using *18S rRNA* as the internal control. All data are presented as mean ± SEM. ** *P* < 0.01 with unpaired *t*-test. **(D)** Relative mRNA levels of *CKS1B* in A549, MIA-PaCa-2, PC3, and U87-MG cells. *CKS1B* expression was examined via qPCR using *18S rRNA* as the internal control. All data are presented as mean ± SEM. *P < 0.05 with unpaired *t*-test, *** *P* < 0.001 with unpaired *t*-test, **** *P* < 0.0001 with unpaired *t*-test. **(E)** RKO/pshCont and RKO/pshNQO1 cells (Left) and MDA-MB-231/pNQO1 and MDA-MB-231/pCont cells (Right) were transfected with pCKS1B promoter-*luc* or control pRL-*luc*. After 4 h, cells were washed with PBS and incubated with the appropriate medium for 48 h. Luciferase activity was normalized with that of *Renilla* (mean ± SEM). *** *P* < 0.001 with unpaired *t*-test, **** *P* < 0.001 with unpaired *t*-test. **(F-G)** RKO/pshCont cells **(F)** and MDA-MB-231/pNQO1 cells **(G)** were transfected with siCont and pCont or siCKS1B and pCont, and RKO/pshNQO1 cells **(F)** or MDA-MB-231/pCont cells **(G)** were transfected with pCont and siCont or pCKS1B and siCont. After 48 h of incubation, cells were synchronized via double-thymidine blocking, released for 9 h, and harvested. The indicated protein levels (Left), cell distribution (Mid) and CDK1 activities (Right) were analyzed using immunoblot analysis, flow cytometry and CDK1 kinase assay, respectively. All data are presented as mean ± SEM. *** *P* < 0.001 with ANOVA, **** *P* < 0.001 with ANOVA.

We further focused on the mechanisms underlying the effects of NQO1 on cancer cell cycle progression at the G2/M phase, initially examining whether NQO1 modifies the transcriptome associated with the cell cycle at the G2/M phase. To this end, RNA was isolated from RKO/pshCont and RKO/pshNQO1 cells and their transcriptomes were analyzed using microarray hybridization. Overall, we identified seven probes associated with cell cycle progression that were downregulated more than two-fold (*P*-value < 0.01) in RKO/pshNQO1 cells compared with RKO/pshCont cells (Figure 3A). In view of the previous finding that CKS1 (encoded by *CKS1B*) is associated with cell cycle progression from G2 phase to M phase 50, we explored the pathway by which NQO1 regulates *CKS1B* to promote progression from G2 phase to M phase in cancer cells. An analysis of *CKS1B* mRNA and CKS1 protein levels in RKO/pshCont and RKO/pshNQO1 cells showed that NQO1 depletion induced a decrease in *CKS1B* mRNA and CKS1 protein expression in RKO cells (Figure 3B and Figure S5A), validating microarray data. Conversely, overexpression of NQO1 enhanced *CKS1B* and CKS1 expression in MDA-MB-231 cells (Figure 3C and Figure S5B). Similar NQO1-mediated *CKS1B* induction was observed in other cancer cell lines, including A549 (lung), MIA PaCa-2 (pancreas), PC3 (prostate), and U87-MG (brain) cell lines (Figure 3D and Figure S5C). To further ascertain the involvement of NQO1 in *CKS1B* mRNA stability, we treated cells for 2 h with 5 μg/mL actinomycin D, which blocks *de novo* mRNA synthesis. As shown in Figure S6, *CKS1B* mRNA stabilization was not associated with NQO1 expression. We further investigated whether NQO1 regulates transcription of *CKS1B* mRNA using a* CKS1B* promoter reporter plasmid (pCKS1B promoter-*luc*). For these experiments, cells were transfected with either pCKS1B promoter-*luc* or control pRL-*luc*. Notably, luciferase activity was significantly inhibited in NQO1-knockdown RKO (RKO/pshNQO1) cells, whereas NQO1-overexpressing MDA-MB-231 cells exhibited elevated luciferase activity relative to NQO1-deficient MDA-MB-231 cells (Figure 3E). To establish whether NQO1-induced CKS1 regulates cancer cell cycle progression at G2/M phase, we transfected NQO1-overexpressing cancer cells with siCont and pCont or siCKS1B and pCont, and NQO1-deficient cancer cells with pCont and siCont or pCKS1B and siCont. After incubating for 48 h, cells were synchronized at the G1/S phase boundary using a double-thymidine block, released for 9 h and harvested, after which protein levels (Figure 3F,G, left panel) as well as cell distribution (Figure 3F,G, middle panel) and CDK1 kinase activities (Figure 3F,G, right panel) were analyzed. CKS1-knockdown in NQO1-expressing cancer cells resulted in an increased proportion of cells at G2/M phase relative to transfection with siCont (Figure 3F,G, middle panel), whereas overexpression of CKS1 decreased the proportion of cells at G2/M phase compared to NQO1-deficient RKO/pshNQO1 and MDA-MB-231/pCont cells (Figure 3F,G, middle panel). Consistent with this, transfection with siCKS1B led to increased CDK1 kinase activity in NQO1-expressing cancer cells relative to siCont-transfected cells (Figure 3F,G, right panel). Furthermore, overexpression of CKS1 in NQO1-deficient cancer cells induced a decrease in CDK1 kinase activity compared to that in cells transfected with pCont (Figure 3F,G, right panel). Our finding that NQO1 regulates *CKS1B* mRNA transcription supports the theory that NQO1 is a critical component of CKS1-mediated cell cycle progression at the G2/M phase in cancer cells.

### NQO1-mediated c-FOS regulates *CKS1B* expression

**Figure 4:**
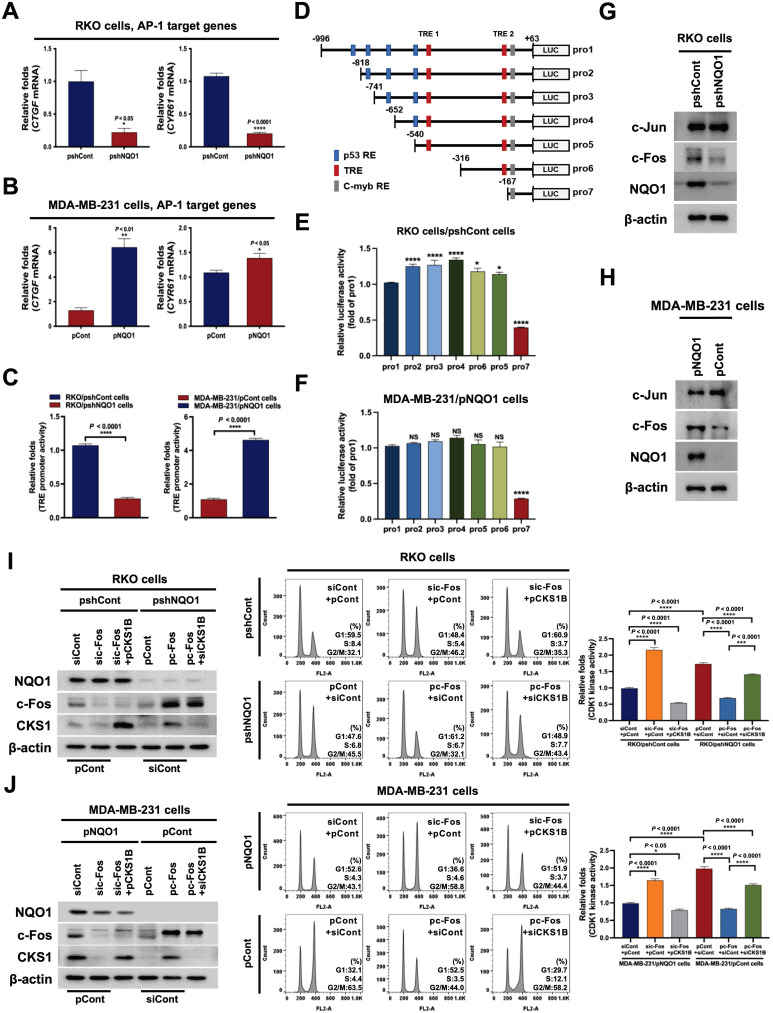
** NQO1-mediated c-FOS regulates *CKS1B* expression. (A-B)** Relative mRNA levels of *CTCF* and *CYR61* in RKO/pshCont and RKO/pshNQO1 cells **(A)** and MDA-MB-231/pNQO1 and MDA-MB-231/pCont cells **(B)**. *CTGF* and *CYR61* expression were examined via qPCR using *18S rRNA* as the internal control. All data are presented as mean ± SEM. * *P* < 0.05 with unpaired *t*-test, ** *P* < 0.01 with unpaired *t*-test, **** *P* < 0.0001 with unpaired *t*-test. **(C)** RKO/pshCont and RKO/pshNQO1 cells (Left) and MDA-MB-231/pNQO1 and MDA-MB-231/pCont cells (Right) were transfected with pTRE-*luc* or the transfection control pRL-*luc*. After 4 h, cells were washed with PBS and incubated with the appropriate medium for 48 h. Luciferase activity was normalized to that of *Renilla* (mean ± SD). **** *P* < 0.001 with ANOVA. **(D)** Deletion constructs of the *CKS1B* promoter generated for the promoter assay. **(E-F)** RKO/pshCont cells **(E)** and MDA-MB-231/pNQO1 cells **(F)** were transfected with the reporter plasmid indicated in **(D)** and the transfection control pRL-*luc*. After 4 h, cells were washed with PBS and incubated with the appropriate medium for 48 h. Luciferase activity was normalized to that of *Renilla* (mean ± SEM). * *P* < 0.05 with ANOVA, ** *P* < 0.01 with ANOVA, **** *P* < 0.001 with ANOVA. NS indicates no significance.** (G-H)** Expression of c-Fos and c-Jun in RKO/pshCont and RKO/pshNQO1 cells **(G)** and MDA-MB-231/pNQO1 and MDA-MB-231/pCont cells **(H)**. Whole-cell lysates were analyzed via immunoblotting for c-Fos, c-Jun, NQO1, and β-actin. **(I-J)** RKO/pshCont cells **(I)** and MDA-MB-231/pNQO1 cells **(J)** were transfected with siCont and pCont, sic-Fos and pCont or sic-Fos and pCKS1B, and RKO/pshNQO1 cells **(I)** or MDA-MB-231/pCont cells **(J)** were transfected with pCont and siCont, pc-Fos and siCont or pc-Fos with siCKS1B. After 48 h incubation, cells were synchronized using double-thymidine blocking, released for 9 h, and subsequently harvested. The indicated protein levels (Left), cell distribution (Mid) and CDK1 activities (Right) were analyzed via immunoblot, flow cytometry and CDK1 kinase assays, respectively. All data are presented as mean ± SEM. ** *P* < 0.01 with ANOVA, **** *P* < 0.001 with ANOVA.

On the basis of the results obtained, we hypothesized that NQO1 regulates one or more transcription factor(s) to promote *CKS1B* expression, since NQO1 itself does not function as a transcription factor. To test this, we examined the effects of NQO1 on transcription factors associated with *CKS1B* expression, identified using a publicly available database. In addition, we employed a microarray analysis to determine the expression levels of transcription factor target genes and used the GeneCards Human Gene Database to analyze binding sites for the transcription factors AP-1, c-Myb, c-Rel, HOXA5, MAZR, p53, p73, Pax-4a and STAT3 in the *CKS1B* promoter sequence. Microarray data showed that NQO1 knockdown (RKO/pshNQO1 cells) induced significant downregulation of the AP-1 (activating protein-1) target genes, *CTGF* and *CYR61*, compared with the corresponding levels in RKO/pshCont cells, an effect that was further confirmed by quantitative polymerase chain reaction (qPCR) analysis (Figure 4A). Conversely, in MDA-MB-231 cells overexpressing NQO1, levels of AP‑1 target genes were increased (Figure 4B). To validate these results, cells were transfected with an asymmetrical palindromic AP-1 binding site (TRE)-containing reporter plasmid (pTRE-*luc*) or transfection control (pRL-*luc*). TRE-mediated transcriptional activity was suppressed in NQO1-knockdown (RKO/pshNQO1) cells, whereas overexpression of NQO1 in MDA-MB-231 cells led to elevated TRE-*luc* activity compared with NQO1-deficient MDA-MB-231 cells (Figure 4C). NQO1 regulation of AP-1-mediated CKS1 expression in cancer cells was further demonstrated by analyzing reporter activity of a series of deletion constructs of a *CKS1B* promoter containing two TREs in transient transfection assays (Figure 4D). The luciferase reporter constructs pro-1 to -7 represent 5'-ends corresponding to nucleotide positions -996, -818, -741, -652, -540, -316, and -167 respectively, from position +63. The promoter activities of pro-1 to -6 were markedly increased in RKO/pshCont and MDA-MB-231/pNQO1 cells, whereas the activity of pro-7 was not affected (Figure 4E,F), indicating that the sequence between -316 and -167 is required for NQO1-mediated TRE-induced *CKS1B* expression. To confirm these results, we constructed TRE deletion mutants by site-directed mutagenesis using pro-1 as a template. As shown in Figure S7, deletion of TRE2 led to decreased luciferase activity.

To further confirm these results, we investigated c-Fos binding to TRE2 on the *CKS1B* promoter using chromatin immunoprecipitation (ChIP) assays. To this end, DNA from RKO/pshCont cells and MDA-MB-231/pNQO1 cells incubated for 16 h was crosslinked, extracted, and incubated with anti-c-Fos antibody or control antibody (anti-IgG). c-Fos/DNA complexes were immunoprecipitated and crosslinking was reversed, followed by PCR amplification targeting TRE2 on *CKS1B*. Complexes immunoprecipitated with the anti-c-Fos antibody generated a PCR band, confirming association with TRE2 in the *CKS1B* promoter, whereas those obtained with the control antibody produced no PCR bands (Figure S8). Previous studies suggested that c-Jun binds DNA as a functional homodimer 51. Because of distinct amino acid interactions within leucine repeat structures, c-Jun/c-Fos heterodimers are thermodynamically more stable than c-Jun homodimers 52. Here, we further focused on determining the components of the AP-1 family affected by NQO1. Proteasomal degradation of c-Fos, and thus its protein stability, is reported to be regulated by NQO1 39. Consistent with earlier findings, NQO1 knockdown suppressed c-Fos expression in RKO/pshNQO1 cells; conversely, NQO1 overexpression in MDA-MB-231 cells had the opposite effect (Figure 4G,H). In addition, inhibition of c-Fos led to a decrease in TRE-*luc* activity regardless of NQO1 expression status (Figure S9).

Next, we investigated whether NQO1-induced c-Fos regulates CKS1 expression and cell cycle progression at the G2/M phase in cancer cells. To this end, NQO1-expressing cancer cells were transfected with siCont and pCont, sic-Fos (c-Fos knockdown) and pCont, or sic-Fos and pCKS1B (CKS1B overexpression), and NQO1-deficient cancer cells were transfected with pCont and siCont, pc-Fos and siCont, or pc-Fos (c-Fos overexpression) and siCKS1B (CKS1B knockdown). After incubating for 48 h, cells were synchronized at the G1/S phase boundary using a double-thymidine block, then released for 9 h, harvested, and analyzed for levels of the indicated proteins (Figure 4I,J, left panel), cell distribution (Figure 4I,J, middle panel), and CDK1 kinase activity (Figure 4I,J, right panel). siRNA-mediated knockdown of c-Fos (sic-Fos transfection) increased the proportion of cells at G2/M phase in NQO1-expressing cancer cells compared with that in the siCont-transfected group (Figure 4I,J, middle panel), an effect that was rescued by reintroduction of CKS1B (Figure 4I,J, middle panel). Conversely, c-Fos overexpression decreased the proportion of cells at G2/M phase compared with that in NQO1-deficient RKO/pshNQO1 and MDA-MB-231/pCont cancer cells (Figure 4I,J, middle panel), and this effect was mitigated by knockdown of CKS1B (Figure 4I,J, middle panel). siRNA-mediated knockdown of c-Fos in NQO1-expressing cancer cells increased CDK1 kinase activity, whereas overexpression of CKS1B decreased c-Fos-mediated enhancement of CDK1 kinase activity in these cells (Figure 4I,J; right panel). In addition, overexpression of c-Fos in NQO1-deficient cancer cells suppressed CDK1 kinase activity, an effect that was enhanced by knockdown of CKS1B (Figure 4I,J; right panel). Taken together, our results suggest that NQO1 regulates *CKS1B* mRNA transcription and cell cycle progression at the G2/M phase in cancer cells through induction of c-Fos expression.

### NQO1 increases c-Fos stability

**Figure 5:**
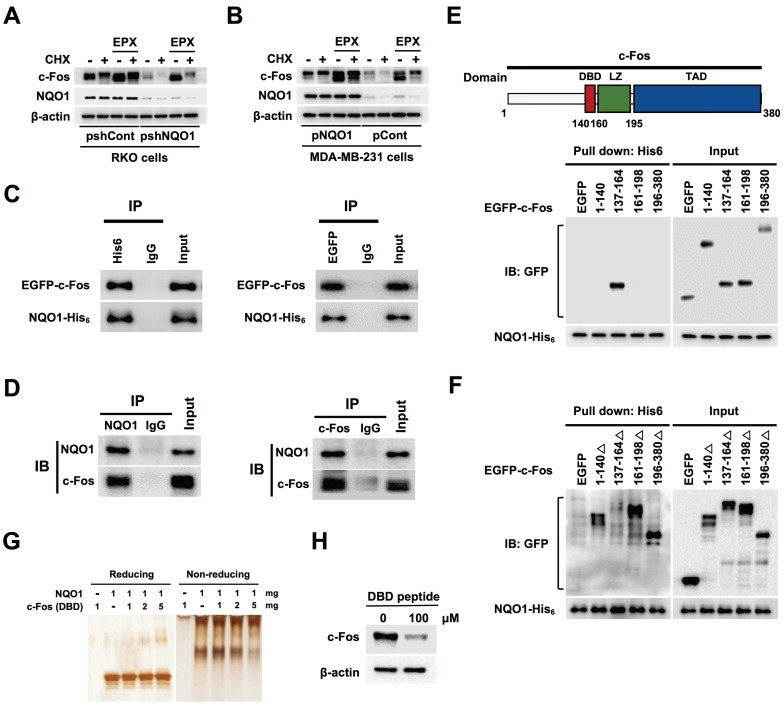
** NQO1 increases c-FOS stability. (A-B)** RKO/pshCont and RKO/pshNQO1 cells **(A)** and MDA-MB-231/pNQO1 and MDA-MB-231/pCont cells **(B)** were incubated with or without EPX for 1 h in the presence or absence of CHX. Whole cell lysates were immunoblotted for c-Fos, NQO1, and β-actin. **(C)** MDA-MB-231 cells were transfected with pNQO1-myc-His_6_ and pEGFP-c-Fos. Whole-cell extracts were immunoprecipitated with anti-His_6_, anti-GFP and anti-IgG (negative control) and analyzed by immunoblotting with anti-NQO1 anti-c-Fos antibodies. **(D)** Whole-cell extracts of RKO/pshCont cells were immunoprecipitated with anti-NQO1, anti-c-Fos and anti-IgG (negative control) and analyzed by immunoblotting with anti-NQO1 anti-c-Fos antibodies. **(E)** (Upper panel) Illustration of domains of c-Fos. DBD, LZ, and TAD represent DNA-binding domain, leucine zipper domain and transcription activation domain, respectively. (Lower panel) MDA-MB-231 cells were transfected with pNQO1-myc-His_6_ and pEGFP-c-Fos expressing deletion constructs, subjected to Ni-NTA bead-based pulldown assays and analyzed by immunoblotting with anti-NQO1 and anti-GFP antibodies. **(F)** MDA-MB-231 cells were transfected with pNQO1-myc-His_6_ and pEGFP-c-Fos expressing deletion constructs, subjected to Ni-NTA bead-based pulldown assays and analyzed by immunoblotting with anti-NQO1 and anti-c-GFP antibodies. **(G)** Binding assay of NQO1 and DBD of c-Fos. Samples were reacted and subjected to electrophoresis using reducing or non-reducing SDS-PAGE and silver staining. **(H)** RKO/pshCont cells were treated with DBD peptide. After 48 h of incubation, whole cell lysates were immunoblotted for c-Fos, and β-actin.

A previous study supports a role for NQO1 as a regulator of c-Fos protein stability 39. Here, using the proteasome inhibitor epoxomicin (EPX) with concurrent inhibition of *de novo* protein synthesis with cycloheximide (CHX), we established the stabilizing effects of NQO1 on c-Fos protein, demonstrating that NQO1 stabilized c-Fos by inhibiting its proteasomal degradation (Figure 5A,B). On the basis of an earlier report that NQO1 binds to c-Fos in the cytosol 39, we further assessed whether NQO1 physically associates with c-Fos by performing c-Fos co-immunoprecipitation (co-IP), Ni-NTA bead-based pull-down assays, and immunofluorescence staining. Results of co-IP assays clearly supported binding of NQO1 to c-Fos (Figure 5C,D). Consistent with a previous report 39, we observed that NQO1 and c-Fos coexist in the cytoplasm (Figure S10). NQO1 has been shown to interact with the leucine zipper domain of c-Fos 39. To identify the binding motifs of c-Fos that interact with NQO1 (Figure 5C,D), we generated various c-Fos deletion mutants linked to an N-terminal EGFP fusion protein. As shown in Figure 5E, NQO1 strongly bound to the c-Fos domain sequence comprising residues 137-164, consistent with the interpretation that residues at positions 140-160 of c-Fos containing the DNA-binding domain (DBD) are essential for interactions with NQO1. Pull-down assays performed using additionally generated c-Fos deletion mutants incorporating residues 1-140, 137-164, 161-198, and 196-380 reinforced this interpretation, demonstrating that NQO1 did not bind deletion mutants of c-Fos lacking residues 137-164 (Figure 5F). To confirm the precise binding sequence, we performed binding assays using NQO1 protein and a peptide containing the DBD (residues 137-164) in which NQO1 protein and DBD peptide were allowed to react, followed by electrophoresis on reducing and non-reducing gels with subsequent silver staining. Notably, the migration of NQO1 in the non-reducing gel was increasingly retarded with increasing concentrations of DBD peptide (Figure 5G). From these results, we hypothesized that DBD peptide would bind to NQO1 and affect the decrease in endogenous c-Fos protein stability. To test this, we treated RKO/pshCont cells with DBD peptide and analyzed c-Fos expression using immunoblot analysis. As shown in Figure 5H, 100 μM DBD peptide decreased expression of endogenous c-Fos in cancer cells. Collectively, our data provide evidence that NQO1 physically interacts with the DBD domain of c-Fos and enhances c-Fos protein stability.

### NQO1-mediated *CKS1B* expression increases radioresistance in cancer cells

**Figure 6:**
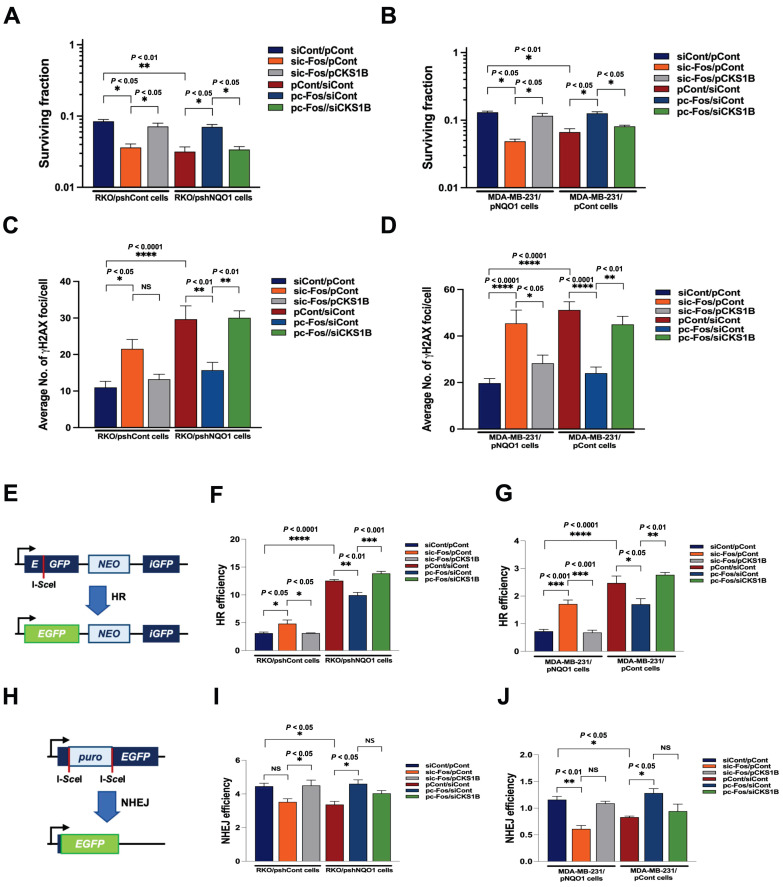
** NQO1-mediated CKS1B expression increases radioresistance in cancer cells. (A-B)** RKO/pshCont cells **(A)** and MDA-MB-231/pNQO1 cells **(B)** were transfected with siCont and pCont, sic-Fos and pCont or sic-Fos and pCKS1B, and RKO/pshNQO1 cells **(A)** or MDA-MB-231/pCont cells **(B)** were transfected with pCont and siCont, pc-Fos and siCont or pc-Fos and siCKS1B. After 48 h of incubation, various quantities of cells were plated on T25 flasks, cultured for 16 h, and irradiated with 0 or 4 Gy, followed by culture for 14 days. Cells in colonies were fixed in 95% methanol, stained with 0.5% crystal violet, and the number of colonies (≥50 cells/colony) from triplicate dishes counted. Mean colony numbers were plotted relative to those formed by untreated cells (mean ± SEM). * *P* < 0.05 with ANOVA, ** *P* < 0.01 with ANOVA. **(C-D)** RKO/pshCont cells **(C)** and MDA-MB-231/pNQO1 cells **(D)** were transfected with siCont and pCont, sic-Fos and pCont or sic-Fos and pCKS1B, and RKO/pshNQO1 cells **(C)** or MDA-MB-231/pCont cells **(D)** were transfected with pCont and siCont, pc-Fos and siCont or pc-Fos and siCKS1B. After 48 h of incubation, various quantities of cells were plated on an eight-well chamber slide, cultured for 16 h, and irradiated with 0 or 4 Gy, followed by culture for 24 h. Cells were fixed with 3.7% PFA and immunofluorescence performed using anti-phospho histone H2AX. γH2AX foci were quantified. All data are presented as mean ± SEM. * *P* < 0.05 with ANOVA, ** *P* < 0.01 with ANOVA. **** *P* < 0.0001 with ANOVA. NS indicates no significance. **(E)** Illustration of the HR repair assay system. **(F-G)** pCBASceI-transfected RKO/pshCont cells **(F)** and MDA-MB-231/pNQO1 cells **(G)** were transfected with siCont and pCont, sic-Fos and pCont or sic-Fos and pCKS1B, and pCBASceI-transfected RKO/pshNQO1 cells **(F)** or MDA-MB-231/pCont cells **(G)** were transfected with pCont and siCont, pc-Fos and siCont or pc-Fos and siCKS1B. After 24 h of incubation, cells were transfected with pGCGFP. Two days after transfection, cells were subjected to flow cytometry analysis (mean ± SEM). * *P* < 0.05 with ANOVA, ** *P* < 0.01 with ANOVA. *** *P* < 0.001 with ANOVA, **** *P* < 0.0001 with ANOVA. **(H)** Illustration of the NHEJ repair assay system. **(I-J)** pCBASceI-transfected RKO/pshCont cells **(I)** and MDA-MB-231/pNQO1 cells **(J)** were transfected with siCont and pCont, sic-Fos and pCont or sic-Fos and pCKS1B, and pCBASceI-transfected RKO/pshNQO1 cells **(I)** or MDA-MB-231/pCont cells **(J)** were transfected with pCont and siCont, pc-Fos and siCont or pc-Fos and siCKS1B. After a 24 h incubation period, cells were transfected with pimEJ5GFP. Two days after transfection, cells were subjected to flow cytometry analysis (mean ± SEM). * *P* < 0.05 with ANOVA, ** *P* < 0.01 with ANOVA. NS indicates no significance.

The radiation sensitivity of cells is dependent on which phase of the cycle cells are in. Specifically, cells are most sensitive to radiation in G2/M phase, less sensitive in G1 phase, and least sensitive during the latter part of S phase 53. Accordingly, we sought to determine whether inhibition of NQO1-induced c-Fos/CKS1 expression leads to increased radiosensitivity through accumulation of cancer cells at the G2/M phase by assessing clonogenic survival; quantifying γH2AX foci, a marker of DNA double-strand breaks (DSBs) and activation of the DNA damage response; and performing homologous recombination (HR) and non-homologous end joining (NHEJ) assays. Clonogenic survival assays showed that knockdown of NQO1 in RKO cells (RKO/pshNQO1) dramatically enhanced radiosensitivity (Figure 6A), whereas ectopic expression of NQO1 in NQO1-deficient MDA-MB-231 cells led to increased radioresistance (Figure 6B). To confirm these findings, we transfected NQO1-expressing cancer cells with siCont and pCont, sic-Fos and pCont or sic-Fos and pCKS1B, and NQO1-deficient cancer cells with pCont and siCont, pc-Fos and siCont or pc-Fos and siCKS1B. In NQO1-expressing cells, sic-Fos transfection increased radiosensitivity, whereas overexpression of CKS1 in sic-Fos-transfected cancer cells led to recovery of radioresistance (Figure 6A,B). Overexpression of c-Fos in NQO1-deficient cancer cells enhanced radioresistance, an effect that was blunted by siRNA-mediated knockdown of CKS1B (Figure 6A,B). Consistent with clonogenic survival data, a quantitative analysis of γH2AX foci in cancer cells revealed that ionizing radiation increased DSBs in cancer cells deficient for NQO1/c-Fos/CKS1 signaling (Figure 6C,D and Figure S11). Cells utilize two major pathways, specifically, NHEJ and HR, to repair DSBs 54. The DSB repair pathway of choice is a tightly regulated process influenced by many factors, such as cell cycle phase and DNA end resection 54. NHEJ can function in all phases of the cell cycle, but is most active in the G1 phase, whereas HR is highly active in S and G2/M phases 54.

To determine whether HR and NHEJ pathways are affected by NQO1/c-Fos/CKS1 signaling, we performed HR and NHEJ reporter assays 54. In these reporter assay systems, I-SceI endonuclease induces DSBs in a reporter plasmid that can only be repaired through HR and NHEJ, and successful repair is detected by monitoring EGFP fluorescence 54. We found that inhibition of the NQO1/c-Fos/CKS1 pathway resulted in accumulation of cells at the G2/M phase. Therefore, HR was increased in irradiated cancer cells deficient for NQO1/c-Fos/CKS1 signaling but decreased in cells where this signaling pathway was activated (Figure 6E-G). NHEJ was slightly decreased in irradiated cancer cells deficient for NQO1/c-Fos/CKS1 signaling but was slightly increased in cells where this signaling pathway remained active (Figure 6H-J). These results indicate that NQO1-induced c-Fos/CKS1 expression increases cancer radioresistance through accumulation of cells at the G2/M phase.

### NQO1 is correlated with CKS1 expression and poor prognosis in cancer

**Figure 7:**
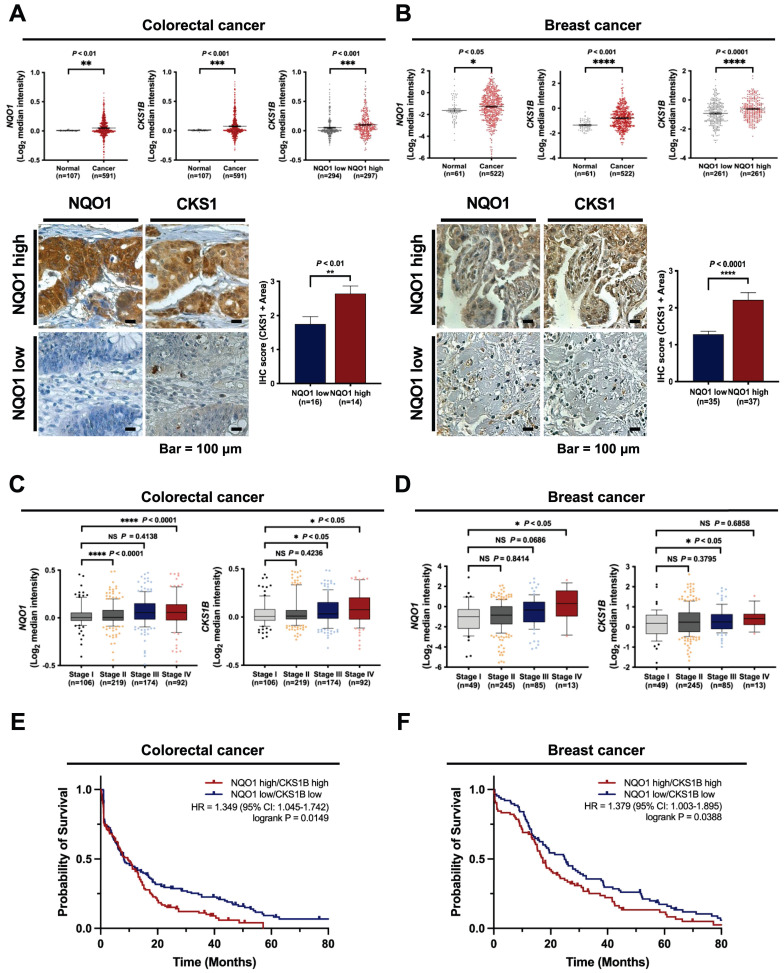
** NQO1 is correlated with CKS1B expression and poor prognosis in cancer. (A-B)** Oncomine analysis of TCGA colorectal cancer **(A)** and TCGA breast cancer **(B)** databases showing elevated *NQO1* (upper left) and *CKS1B* (upper mid) levels in colorectal cancer (n = 591) and breast cancer (522) compared to normal colorectal tissue (n = 107) and breast tissue (n = 61), respectively. * *P* < 0.05 with unpaired *t*-test. ** *P* < 0.01 with unpaired *t*-test. *** *P* < 0.001 with unpaired *t*-test. **** *P* < 0.0001 with unpaired *t*-test. Analysis of CKS1B expression relative to that of NQO1 in the Oncomine database (upper right). *** *P* < 0.001 with unpaired *t*-test. **** *P* < 0.0001 with unpaired *t*-test. Immunohistochemical detection (lower left) of CKS1 under conditions of high-level expression of NQO1 (Colorectal cancer, n=14; Breast cancer, n=37) compared to low-level expression of NQO1 (Colorectal cancer, n=16; Breast cancer, n=35). Positive area scores of NQO1 and CKS1 were determined in the most characteristic areas. The positive area score (lower right) of CKS1 was evaluated from 10 high magnification power fields (×40). Statistical analysis of the average CKS1 positive area score is shown in the right panel (Colorectal cancer, ** *P* < 0.01 with unpaired *t*-test; Breast cancer, **** *P* < 0.0001 with unpaired *t*-test). Bar = 100 μm. **(C-D)** Oncomine analysis of colorectal cancer **(C)** and breast cancer **(D)** showing that elevated NQO1 and CKS1 mRNA levels are correlated with advanced stages of colorectal and breast cancer. **(E)** NQO1 and CKS1 expression correlates with poor survival in colorectal cancer data set. Analysis of the colorectal cancer data set available through Oncomine indicates a significant correlation between the high-level expression of NQO1 and CKS1, and poor survival in the TCGA data set (n = 130 NQO1 high/CKS1 high, n = 151 NQO1 low/CKS1 low; *P* = 0.0149 with log-rank analysis). HR, hazard ratio; CI, confidence interval. **(F)** NQO1 and CKS1 expression correlates with poor survival in breast cancer data set. Analysis of the breast cancer data set available through Oncomine indicates a significant correlation between the high-level expression of NQO1 and CKS1, and poor survival in the TCGA data set (n = 84 NQO1 high/CKS1 high, n = 88 NQO1 low/CKS1 low; *P* = 0.0388 with log-rank analysis). HR, hazard ratio; CI, confidence interval.

Based on the above findings, we investigated the clinical significance of NQO1 and CKS1 expression in cancer cells. The correlations between NQO1 and CKS1 expression and clinicopathological features in cancer patients were determined with the aid of publicly available data sets and immunohistochemical analysis. Analysis of The Cancer Genome Atlas (TCGA) database revealed significant correlation of elevated *NQO1* and *CKS1B* levels (Figure 7A-B). Expression levels of NQO1 and CKS1 were defined by their positive area scores (Figure S12). Immunohistochemical (IHC) analyses of colorectal and breast cancer tissues and cell lines used in this study demonstrated that high NQO1-expressing tumors frequently had IHC scores of 2.5 and 2.1 together with significantly elevated expression of CKS1 (Figure 7A,B). Further assessment of TCGA colorectal cancer and breast cancer databases supported correlations of high *NQO1* and *CKS1B* expression with tumor stage (Figure 7C,D). To evaluate the potential association of *NQO1* and *CKS1B* expression with patient outcomes, we performed a Kaplan-Meier survival analysis using both TCGA colorectal and breast cancer datasets, which revealed a strong correlation of high *NQO1* and *CKS1B* expression with poor prognosis (Figure 7E,F). Collectively, these findings clearly indicate that aberrantly elevated NQO1 expression is associated with poor prognosis in patients with colorectal or breast cancer.

## Discussion

NQO1 is a cytosolic reductase that exerts essential cytoprotective antioxidant effects by catalyzing the two-electron reduction of potentially toxic quinones, thereby preventing cytotoxicity of diverse carcinogens 11-15. The C609T mutant form of NQO1 is associated with a higher risk of tumor development in several human cancer types 55, 56. In addition, upregulation of NQO1 is closely correlated with poor prognosis in breast, colorectal, ovarian, and cervical cancers 3-5. Here, we uncovered a previously unidentified pro-tumorigenic role of NQO1 that is engaged upon an intrinsic increase in CKS1, a central regulatory protein that functions in G1-S and G2-M transition in the cell cycle. Furthermore, we provide evidence that NQO1 physically interacts with the DBD of c‑Fos preventing its proteasomal degradation and thus stabilizing c-Fos protein, which, in turn, promotes CKS1 expression. Given the significant involvement of CKS1 in cancer cell growth, invasion, metastasis and drug resistance 45, our findings offer a possible mechanistic explanation for the association of NQO1 overexpression with poor clinical outcomes in cancer patients.

*NQO1*, together with genes encoding pentose phosphate pathway enzymes, ATP-binding cassette (ABC) transporter and some heme-metabolizing enzymes, is among the targets of nuclear factor (erythroid-derived 2)-like 2 (NRF2), which acts through the proteins encoded by its target genes to protect cells from intracellular and extracellular oxidative stress 57. The stability of NRF2 protein is controlled by KEAP1 (Kelch-like ECH-associated protein 1), a subunit of the Cul3/RBX1 E3 ubiquitin ligase complex, which regulates NRF2 proteasome-mediated degradation and thereby contributes to the maintenance of a low level of NRF2 in the cell 58. NRF2 is constitutively expressed at high levels in human cancers, where it protects against the excessive oxidative stress caused by chemotherapies and radiotherapies 58. This aberrant activation of NRF2 is attributable to somatic mutations in the *KEAP1* or *NRF2* gene or other mechanisms that disrupt the binding of KEAP1 to NRF2 57, 58. NRF2 and KEAP1 status in the cell lines used in this study, and the stimuli that activate NRF2, are summarized in Supplementary Table 1.

Suppression of NQO1 induces growth-inhibitory effects 59. In an earlier study, dicoumarol, a potent inhibitor of NQO1, was shown to suppress formation of pancreatic cancer cell colonies on soft agar 60 and decrease the viability and proliferation rates of HeLa cells 61. Moreover, our group previously showed that an NQO1 deficiency leads to aneuploidy in irradiated cancer cells during mitotic progression 41. NQO1 is also reported to affect mitotic progression through regulation of SIRT2 activity 43. In accord with these findings, the present study showed that knockdown of NQO1 reduced the proliferative ability of RKO colorectal cancer cells (Figure 1A,C). Conversely, ectopic overexpression of NQO1 in NQO1-deficient MDA-MB-231 breast cancer cells led to a marked increase in cell proliferation (Figure 1B,D). Furthermore, we observed whether NQO1 regulates cell cycle progression by regulating the mitotic spindle formation. As shown in Figure S13, it was observed that the presence or absence of NQO1 did not affect the mitotic spindle in cells undergoing mitosis. To extend these previous and current observations to a consideration of cell cycle-dependent aspects of NQO1 function, we synchronized NQO1-deficient or -replete cancer cells (and respective controls) at G1/S and G2/M phases using thymidine block and nocodazole, respectively, and after subsequently releasing cells from block, analyzed cell cycle progression. As shown in Figure 2A and B, an NQO1 deficiency in cancer cells caused a delay in cell cycle progression at the G2/M phase. Levels of cyclin B1 in RKO/pshCont cells (expressing endogenous NQO1) and MDA-MB-231/pNQO1 cells (exogenously expressing NQO1) were increased in S and G2/M phases, respectively, and rapidly decreased in the G1 phase (Figure 2C,D). However, cyclin B1 expression was delayed in NQO1-deficient RKO/pshNQO1 and MDA-MB-231/pCont cells and gradually decreased (Figure 2C,D). Moreover, after cell cycle synchronization using thymidine and release at the G1/S phase, CDK1 kinase activity in NQO1-deficient cancer cells was concurrently increased, as it was in NQO1-expressing cancer cells, but the decrease was delayed (Figure 2E,F). A previous report showed that, in melanoma, NQO1 mediates activation of the NF-κB component p50 through stabilization of BCL3-induced cell cycle progression and proliferation 62. However, the consequences of NQO1-NF-κB interactions in breast cancer are different from those in other cancers 63. In contrast to a previous report that arsenic pollution-induced NRF2/NQO1 signaling regulates cell cycle progression at G1/S in squamous cell carcinoma 64, in the present study, we found that NQO1 regulates cell cycle progression at G2/M phase (Figure 2). Therefore, additional studies are warranted to clarify the role of NQO1 in the complex pathway governing G2/M phase cell cycle progression.

To better understand the involvement of NQO1 in cell cycle progression, we investigated gene expression in RKO/pshCont and RKO/pshNQO1 cells, demonstrating that NQO1 increased transcription of *CKS1B* encoding the CKS1 protein in cancer cells (Figure 3A-D). CKS1 is a major regulatory protein that exerts effects at the G1-S phase and G2-M transition 45. Two CKS proteins, CKS1 and CKS2, have been identified in mammalian cells 46-48. CKS1 is required for SCF^Skp2^-mediated ubiquitination and degradation of p27^kip1^, which is essential for G1/S transition during the cell cycle 65. CKS2 is involved in the first metaphase/anaphase transition in mammalian meiosis, but its precise role is not clear 65. CKS1 has been reported to be highly expressed in many cancer types, including breast cancer, colon cancer, lung cancer, hepatocellular carcinoma, and retinoblastoma 45. In addition, CKS1 has been identified as one of 70 high-risk genes whose expression is inversely proportional to survival of patients diagnosed with multiple myeloma 45. CKS1-depleted cells not only exhibit slower G1 phase progression, they also accumulate at the G2/M phase owing to blockage of mitotic entry due to drastically reduced expression of CDK1 49. CKS1 is additionally reported to regulate S phase entry 44. In the current study, an NQO1 deficiency led to reduced CKS1 expression in cancer cells and delayed cell cycle progression at the G2/M phase. In view of the finding that *CKS1B* is upregulated by NQO1 in cancer cells, we hypothesized that NQO1 promotes *CKS1B* expression in cancer cells through regulation of a transcription factor.

To further identify the transcription factor(s) that mediate *CKS1B* expression in cancer cells, we analyzed gene expression in NQO1-containing (RKO/pshCont) and NQO1-deficient (RKO/pshNQO1) cells. Microarray analyses showed that an NQO1 deficiency led to a significant downregulation of the AP-1 target genes, *CTGF* and *CYR61*, in RKO/pshNQO1 compared with RKO/pshCont cells, an effect confirmed in MDA-MB-231 cells. AP-1, which is linked to cancer, is a dimeric transcription factor composed of proteins belonging to the Jun (c-Jun, JunB, and JunD), Fos (c-Fos, FosB, Fra1, and Fra2), and activating transcription factor (ATF) families 66. The transcriptional activity of AP-1 is regulated by a wide array of cellular stimuli, including growth factors, bacterial and viral infections, cytokines, UV radiation, and cellular stresses 67. AP-1 family members, in particular c-Jun, are highly expressed in invasive cancers and mediate enhanced migration and proliferation 68. Overexpression of c-Fos is correlated with poor prognosis in different cancer types, including human squamous cell lung carcinoma, breast carcinoma, osteosarcoma, oral squamous cell carcinoma, and cutaneous squamous cell carcinoma 69. AP-1 DNA recognition elements (5'-TGAG/CTCA-3') are also known as TREs 70. c-Fos proteins do not form stable dimers but can bind DNA by forming heterodimers with Jun proteins that are thermodynamically more stable than Jun:Jun homodimers 71. To establish whether NQO1 regulates AP-1-mediated CKS1 expression in cancer cells, we analyzed the *CKS1B* promoter, which contains two AP-1-like elements, using a promoter deletion assay and a reporter plasmid. This analysis showed that the sequence between positions -316 and -167 was required for NQO1-mediated AP-1 induction of *CKS1B* expression (Figure 4D-F and Figure S7). A further examination of the potential regulatory effects of NQO1 on c-Fos and c-Jun showed that NQO1 knockdown led to reduced expression of c-Fos, but not c-Jun, in RKO cells (Figure 4G). Conversely, ectopic introduction of NQO1 into NQO1-deficient MDA-MB-231 cells induced an increase in the expression of c-Fos but not c-Jun (Figure 4H). Thus, our data suggest that NQO1 stimulates CKS1 expression through upregulation of c-Fos, which, in turn, regulates cell cycle progression in cancer cells at the G2/M phase (Figure 4I,J).

A previous report by Adler et al. demonstrated that NQO1 physically interacts with newly synthesized c-Fos in the cytoplasm and protects it from degradation until it engages in formation of an AP-1 complex and localizes to the nucleus 39. The subcellular localization of c-Fos is controlled by its import from the cytoplasm to the nucleus and its retention in the nuclear compartment 72. Nuclear localization and retention are dependent on protein domains within c-Fos 72, interactions with Jun family members 73, and the appropriate signaling 44, 75. NQO1 is found mainly in the cytoplasm 76, with a small amount in the nucleus 77. Exogenously overexpressed c-Fos is mostly detected in the nucleus, whereas overexpression of c-Jun or NQO1 results in massive accumulation of c-Fos in the nuclear fraction or cytoplasm, respectively 39. Overexpression of both NQO1 and c-Jun leads to the localization of all accumulated c-Fos exclusively in the nucleus 39. Because binding of NQO1 to c-Fos is severely reduced in the presence of c-Jun 39, reflecting the nuclear localization of c-Jun-bound c-Fos, NQO1-induced c-Fos accumulation increases c-Fos target gene transcription 39. NQO1 has been shown to increase c-Fos protein stability by binding to its leucine zipper domain and inhibiting its proteasome-mediated degradation 39. In this context, NQO1 stabilizes several proteins, including CEBPα, p53, p73 and p33ING1b, by blocking their proteasomal degradation 34-38, 40. c‑Fos and c-Jun contain structurally similar leucine repeats 39. Thus, if NQO1 is involved in increasing c-Fos protein stability via its leucine zipper domain, similar binding to the leucine zipper domain of c-Jun is plausible. However, we observed no changes in c-Jun level regardless of the expression status of NQO1 (Figure 4G,H), leading us to conclude that NQO1 does not bind the leucine zipper domain of c-Jun, an inference further confirmed by Co-IP (Figure S14). In a previous report, the leucine zipper domain of c-Fos containing the DBD was utilized to determine interactions with NQO1 39. In the current study, c‑Fos binding to NQO1 was monitored after separately expressing the leucine zipper domain and DBD of c‑Fos (Figure 5E-G). Collectively, these data provide strong evidence that NQO1 physically interacts with the DBD domain of c-Fos protein and increases its stability. In a previous report, the Fos family proteins, FosB and c-Fos, were shown to control cell cycle progression in fibroblasts through regulation of cyclin D1 78. According to this report, cell cycle progression was completely inhibited in cells lacking FosB (FosB^‑/‑^) and c-Fos (c-Fos^-/-^) genes 78, whereas cell cycle progression was partially inhibited in c-Fos^-/-^ cells heterozygous or homozygous for the FosB gene (i.e., FosB^+/-^ or FosB^+/+^) 78. Another report demonstrated that c-Fos and E2F-mediated induction of cyclin A regulates cell cycle progression in vascular smooth muscle cells 79, indicating that c-Fos acts through different factors to impact cell cycle progression. Therefore, further investigation of the role of NQO1 in the context of the complex relationship between the involvement of CKS1 in cell cycle progression and c-Fos in cancer cells is warranted, an investigation that we plan to undertake in the future. Anti-mitotic therapies targeting the G2/M phase have been considered a prototypical strategy against abnormally proliferating cells 80. G2/M phase inhibitors include anti-microtubule agents, PLK1 inhibitors, AURK inhibitors, survivin inhibitors and CDK inhibitors, and various clinical trials are currently underway 81. Our current finding that the DBD peptide of c-Fos binds to NQO1 is a unique and important piece of information that could aid in the development of new and effective means for targeting the G2/M phase using c-Fos inhibitors. Furthermore, the development of DBD-based peptides that inhibit c-Fos based on our findings will provide important information for the development of peptide-based drugs with fewer side effects.

An NQO1 deficiency in cancer cells leads to a delay in cell cycle progression at the G2/M phase through reduced c-Fos-mediated CKS1 expression (Figures 1-3). Because the radiation sensitivity of cells is dependent on cell cycle phase such that cells are most sensitive to radiation in G2/M phase, less sensitive in G1 phase, and least sensitive during the latter part of S phase 53, we investigated whether NQO1 functionally contributes to increased susceptibility of cancer cells to ionizing radiation. As shown in Figure 6, knockdown of NQO1 induced an increase in DSBs, thereby markedly increasing HR, decreasing NHEJ, and enhancing the radiosensitivity of cancer cells. Conversely, ectopic expression of NQO1 in NQO1-deficient MDA-MB-231 cells increased radioresistance. These observations suggest that this NQO1/c-Fos/CKS1 signaling mechanism provides insights useful for the development of therapeutic strategies targeting the G2/M phase of the cell cycle.

To establish the potential prognostic value of NQO1 in human cancer, we evaluated publicly available colorectal cancer and breast cancer datasets. NQO1 levels in colorectal and breast cancer specimens were significantly higher than those in normal colorectal and breast tissue counterparts 3, 40, and CKS1 was overexpressed in many cancer types, including breast cancer, colon cancer, lung cancer, hepatocellular carcinoma, and retinoblastoma 45. Consistent with previous reports 3,40, we found that expression levels of *NQO1* and *CKS1B* reported in TCGA colorectal and breast cancer datasets were higher in colorectal and breast cancer compared with normal tissues and that *CKS1B* expression was dependent on the level of *NQO1* expression (Figure 7A,B). These results, based on IHC analyses of tissues from colorectal and breast cancer patients, indicate that high expression of NQO1 is associated with high expression of CKS1 (Figure 7A,B). We also found that expression levels of *NQO1* and *CKS1B* mRNA in colorectal cancer were highly increased in tumor stages 3 and 4 compared with tumor stages 1 and 2 (Figure 7C). In breast cancer, *NQO1* expression is highly increased, and expression of *CKS1B* is also increased, albeit to a slightly lesser degree, but in both cases this increase is dependent on tumor stage (Figure 7D). Furthermore, we found that high *NQO1* and *CKS1B* expression is correlated with poor patient prognosis (Figure 7E,F).

**Figure 8:**
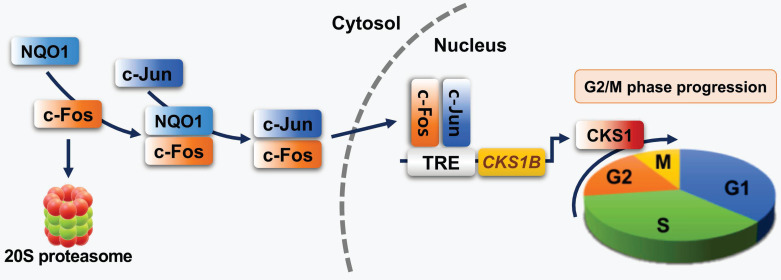
** Schematic model showing how NQO1 regulates cell cycle progression at the G2/M phase in cancer cells.** Newly synthesized c-Fos is degraded by the 20S proteasome. NQO1 physically interacts with c-Fos to inhibit degradation by the 20S proteasome, after which c-Fos associates with c-Jun, forming an AP-1 complex that translocates to the nucleus and binds to the TRE of the *CKS1B* promoter to drive expression of the *CKS1B* gene. Upon translation, CKS1 protein regulates G2/M phase progression of the cell cycle.

In summary, our study demonstrates for the first time that NQO1 induces marked upregulation of CKS1 by stabilizing c-Fos protein (Figure 8) in cancer cells, leading to accelerated cell proliferation. Our findings provide novel insights into the role of NQO1/c-FOS/CKS1 signaling in cancer, highlighting an innovative avenue for anticancer therapeutic strategies targeting this pathway.

## Materials and Methods

### Cell lines and culture conditions

A549 human lung adenocarcinoma epithelial cells, MDA-MB-231 human breast cancer cells, MIA PaCa-2 human pancreatic cancer cells, RKO human colon cancer cells, PC3 human prostate cancer cells, and U87-MG glioblastoma cells were obtained from American Type Culture Collection (ATCC) and cultured in DMEM or RPMI medium. Cells were incubated at 37^o^C in a 5% CO_2_-containing humidified incubator unless otherwise specified. All cell lines were tested for the presence of mycoplasma via PCR.

### *NQO1* and *CKS1B* gene expression in human cancers

Correlations between *NQO1* and *CKS1B* gene expression were analyzed using TCGA available from Oncomine (Compedia Biosciences, http://www.oncomine.org/). High and low expression groups were defined as those with *NQO1* and *CKS1B* levels above and below the mean value, respectively.

### CDK1 kinase assay

Cells were washed twice with PBS (pH 7.2), resuspended in PBS (pH 7.2) containing an inhibitor cocktail (Roche Applied Science), sodium orthovanadate, and sodium fluoride, sonicated four times using 10-second pulses on ice, and centrifuged at 14,000 g for 20 minutes. The supernatant fractions were collected, transferred into microcentrifuge tubes, and stored at -80^o^C until use in the CDK1 kinase assay. CDK1 kinase activity was analyzed using a specific CDK1 kinase assay kit (#79597, BPS Bioscience) according to the manufacturer's instructions.

### RNA preparation and microarray

Total RNA was extracted from RKO/pshCont and RKO/pshNQO1 cells using TRIzol reagent (Invitrogen) in keeping with the manufacturer's instructions. The quality and quantity of RNA was assessed with an Agilent 2100 bioanalyzer system (Agilent Technologies). Gene expression was analyzed with GeneChip® Affymetrix Primeview array (Affymetrix) composed of over 530,000 probes representing ~20,000 well-characterized human genes. For each gene, eleven pairs of oligonucleotide probes were synthesized *in situ* on the arrays. Biotinylated cRNA was prepared from 500 ng total RNA according to the standard Affymetrix protocol (Expression Analysis Technical Manual, 2001, Affymetrix). Following fragmentation, 12 μg RNA was hybridized for 16 h at 45^o^C on a GeneChip Human Genome Array. GeneChips were washed and stained in the Affymetrix Fluidics Station 450 and scanned using the Affymetrix GeneChip Scanner 3000 7G. Data were analyzed via Robust Multi-array Analysis (RMA) using Affymetrix default analysis settings and global scaling as the normalization method. The trimmed mean target intensity of each array was arbitrarily set to 100. Normalized and log-transformed intensity values were subsequently analyzed using GeneSpring GX 12.6 (Agilent Technologies). Fold change filters included the requirement for genes to be present in at a level of 200% of control for upregulated genes and lower than 50% of control for downregulated genes. Through hierarchical clustering, data were clustered into groups that behaved similarly across experiments using GeneSpring GX 12.6.1 (Agilent Technologies). The clustering algorithm was Euclidean distance average linkage.

### Chemicals and antibodies

Cycloheximide (CHX) and epoxomicin (EPX) were purchased from Calbiochem (Merck KGaA). Sodium orthovanadate, sodium fluoride, β-glycerophosphatate, thymidine, hyroxyurea, and nocodazole were acquired from Sigma-Aldrich, along with trifluoroacetic acid (TFA), ninhydrin, piperidine and acetic anhydride. N,N´-dimethylformamide (DMF), triisopropylsilane (TIS) and HPLC solvents were purchased from Acros Organics and diisopropylethyl amine (DIPEA) and 3,6-dioxa-1,8-octanedithiol from TCI. Rink Amide MBHA resin was acquired from Advanced Chem Tech. 1-Hydroxybenzotriazole (HOBt) and o-(benzotriazol-1-yl)-N,N,N´,N´-tetramethyluronium hexafluorophosphate (HBTU) and N-α-Fmoc-protected amino acids (Novabiochem) were used as received. Antibodies were obtained from the following sources: anti-cyclin B1 (1:1000; sc-245, Santa Cruz Biotechnology), anti-NQO1 (1:2,000; 39-3700, Invitrogen), anti-β-actin (1:5000; A5316, Sigma-Aldrich), anti-CDK1 (1:1000; sc-54, Santa Cruz Biotechnology), anti-CKS1 (1:500; #36-6800, Invitrogen), anti-c-Fos (1:1000; #2250S, Cell Signaling Technology), anti-c-JUN (1:1000; #9165, Cell Signaling Technology), anti-His_6_ (1:1,000; 11 911 416 001, Roche Applied Science), and anti-GFP (1:1,000; 11 814 460 001, Roche Applied Science). Secondary antibodies were obtained from the following sources: anti-rabbit Alexa^TM^ Fluor 488 (1:100; A11008, ThermoFisher Scientific), anti-rabbit Alexa^TM^ Fluor 594 (1:100; A11012, ThermoFisher Scientific), anti-mouse HRP (1:2,000; #7076S, Cell Signaling Technology), and anti-rabbit HRP (1:2,000; #7074S, Cell Signaling Technology). The antibodies used as negative controls for immunoprecipitation included normal mouse IgG (sc-2025, Santa Cruz Biotechnology) and normal rabbit IgG (sc-2027, Santa Cruz Biotechnology).

### Construction of plasmids and stable cell lines

To construct a luciferase reporter plasmid encoding *CKS1B* promoter (ppro#-luc), total DNA was extracted from RKO cells using the AccuPrep® Genomic DNA extraction kit (Bioneer). The promoter of *CKS1B* was amplified via PCR with the appropriate primer pairs as follows: pro1, 5'-GGG GTA CCT CCC ACA AAG ATA AAG CTC-3' (forward) and 5'-GGA ATT CTC ATT TCT TTG GTT TCT TGG G-3' (reverse); pro2, 5'-GGG GTA CCT CAA CAA ATT CGA ATC GTT C-3' (forward) and 5'-GGA ATT CTC ATT TCT TTG GTT TCT TGG G-3' (reverse); pro3, 5'-GGG GTA CCC CGA GGA TCG TTG CTA G-3' (forward) and 5'-GGA ATT CTC ATT TCT TTG GTT TCT TGG G-3' (reverse); pro4, 5'-GGG GTA CCG GAC TTC CAG AAA AAC TGG-3' (forward) and 5'-GGA ATT CTC ATT TCT TTG GTT TCT TGG G-3' (reverse); pro5, 5'-GGG GTA CCG TCT CAG ACT TAT AAA TGA AG-3' (forward) and 5'-GGA ATT CTC ATT TCT TTG GTT TCT TGG G-3' (reverse); pro6, 5'-GGG GTA CCT ATT ACA CTC ACT TCC GGC-3' (forward) and 5'-GGA ATT CTC ATT TCT TTG GTT TCT TGG G-3' (reverse); pro7, 5'-GGG GTA CCG CTC GTT CTT GAG AAG CG-3' (forward) and 5'-GGA ATT CTC ATT TCT TTG GTT TCT TGG G-3' (reverse); △TRE1, 5'-AAG TCA AGA CTC ACC AGG-3' (forward) and 5'-CTT CCG GTC TTC ATT TAT AAG-3' (reverse); △TRE2, 5'-CGA GCT CCG CTC GTT CTT GAG AAG C-3' (forward) and 5'-CGA GCT CAA AGT AGG CGT CTT ATT GGC-3' (reverse); TRE1, 2△ 5'-CGA GCT CCG CTC GTT CTT GAG AAG C-3' (forward) and 5'-CGA GCT CCT TTC ACC CCG GAA CCC-3' (reverse) (Bioneer). PCR products were digested with the appropriate restriction enzymes and directly ligated into pGL3-basic (Invitrogen). To construct plasmids expressing NQO1 and the EGFP-c-FOS fusion protein, total RNA was obtained from RKO cells using TRIzol reagent (Invitrogen) and cDNA generated using SuperScriptTM III Reverse Transcriptase (Invitrogen). The open reading frames (ORF) of NQO1 and c-FOS were amplified via PCR with the appropriate primers as follows: NQO1, 5'-GGG GTA CCA TGG TCG GCA GAA GAG CAC-3' (forward) and 5'-CCG CTC GAG TTT TCT AGC TTT GAT CTG G-3' (reverse); c-FOS, 5'-CGG GAT CC ATG ATG TTC TCG GGC TTC AAC-3' (forward) and 5'-GGA ATT C CAG GGC CAG CAG CGT GG-3' (reverse); aa1-140, 5'-GGA ATT CTA TGA TGT TCT CGG GCT TCA AC-3' (forward) and 5'-GGG GTA CCC CTT TTC TCT TCT TCT TCT GG-3' (reverse); aa137-164, 5'-GGA ATT CTG AAG AGA AAA GGA GAA TCC G-3' (forward) and 5'-GGG GTA CCT GTA TCA GTC AGC TCC CTC-3' (reverse); aa161-198, 5'-GGA ATT CTC TGA CTG ATA CAC TCC AAG-3' (forward) and 5'-GGG GTA CCT GCC AGG ATG AAC TCT AG-3' (reverse); aa196-380, 5'-GGA ATT CTA TCC TGG CAG CTC ACC G-3' (forward) and 5'-GGG GTA CCC AGG GCC AGC AGC GTG-3' (reverse) (Bioneer). Amplified products were digested with restriction enzymes and directly ligated into pCDNA3.1-myc-His_6_ (Invitrogen) or pEGFP-c1 (Clontech) vectors for cloning. The cloned plasmids were analyzed via restriction digestion and DNA sequencing (Bionics). To construct stable cell lines, cells were seeded at a density of 5 × 10^4^ cells per well in 24-well plates and transfected with 50 μl mixture containing 1 μg pCDNA3.1-NQO1-myc-His_6_ (Invitrogen) or pshNQO1 (Qiagen) along with TurboFect in vitro transfection reagent (Fermentas). pCDNA3.1-myc-His_6_ (pCont) and pshCont (Qiagen) were utilized as controls. Transfected cells were selected with 1 mg ml-1 G418 or 1 μg ml-1 puromycin (Duchefa Biochemie) for 1 week and maintained in DMEM or RPMI-1640 containing 0.5 mg ml-1 G418 or 0.3 μg ml-1 puromycin during the experiments.

### Reporter assays

Cells (5 × 10^4^) were seeded into 25 cm^2^ flasks, incubated overnight, and co-transfected with 50 μl mixture containing 1 μg 3 × AP1pGL3 (plasmid #40342, Addgene), ppro#-*luc* or pCKS1B promoter-*luc* and 0.01 μg pRL-*luc* (transfection control; Promega) using the TurboFect *in vitro* transfection reagent (Fermentas). After 4 h, cells were washed with PBS and incubated with the appropriate medium for 48 h. Luciferase activity was determined using a luciferase assay system (Promega) and normalized with respect to *Renilla* luciferase activity according to the manufacturer's instructions. Three independent transfections were performed in each case.

### RNA isolation and qPCR

Total RNA was extracted from RKO and MDA-MB-231 cells using TRIzol reagent (Invitrogen) and treated with DNase I (New England Biolabs). Next, total RNA (1 μg) was used for cDNA synthesis with AccuPower RT PreMix (Bioneer, Daejeon, Republic of Korea), and the resulting cDNA amplified via PCR with the following primer pairs: NQO1, 5'-CCC TGC GAA CTT TCA GTA TCC-3' (forward) and 5'-CTT TCA GAA TGG CAG GGA CTC-3' (reverse); overexpressed NQO1, 5'-TTG ACC TAA ACT TCC AGG C-3' (forward) and 5'-TAG AAG GCA CAG TCG AGG CTG-3' (reverse); CTGF, 5'- TCC CGA GAA GGG TCA AGC T-3' (forward) and 5'- TCC TTG GGC TCG TCA CAC A-3' (reverse); CYR61, 5'-TCC TCT GTG TCC CCA AGA AC-3' (forward) and 5'-TCG AAT CCC AGC TCC TTT ACC-3' (reverse) (Bioneer). qPCR was performed using iQ SYBR Green Supermix (2x) (Bio-Rad) and analyzed with the CFX Connect^TM^ Real-Time PCR Detection System (Bio-Rad).

### Synthesis of c-Fos (DBD) peptide

The NQO1-binding c-Fos peptide (Ac-EEKRRIRRER NKMAAAKCRNRRRELT-NH2) was synthesized using Fmoc-chemistry in solid phase peptide synthesis 82. Fmoc-protected amino acids were assembled on Rink Amide MBHA resin. The coupling reaction for each amino acid was conducted using 3-molar excess of the corresponding Fmoc amino acid and coupling reagents in DMF. HBTU (0.2 mmol), HOBt (0.2 mmol), DIPEA (0.4 mmol) and Fmoc-protected amino acids (0.2 mmol) in DMF (2 mL) were added to Rink amide resin (200 mg, 0.1 mmol) and the resulting solution stirred for 1 h at room temperature. After filtration, the resin was washed three times with DMF (3 mL) and methanol (3 mL). The coupling reaction was repeated until no color change was observed in the ninhydrin test. The Fmoc protection group on the resin was subsequently removed by addition of 25% piperidine in DMF. After 15 min stirring, the resin was washed three times with DMF (3 mL) and methanol (3 mL), respectively. Following completion of solid-phase synthesis, the peptide was deprotected and cleaved from the resin by treatment with a mixture of TFA/TIS/3,6-dioxa-1,8-octane-dithiol/H2O (94:1:2.5:2.5, v/v) at room temperature for 4 h. Subsequent to peptide cleavage, the resin was filtered and excess TFA removed in solution. The peptide was obtained by precipitation into cold diethylether at -20ºC. Crude peptide was purified via reverse-phase HPLC (YL9100, Yonung Lin Instruments, Republic of Korea) using a C18 column (Sunfire C18, 4.6 × 150 mm) with buffer A (water with 0.1%, v/v TFA) as the stationary phase and buffer B (acetonitrile with 0.1%, v/v TFA) as the mobile phase. The gradient conditions of the mobile phase were: 5 min at 100% A followed by a linear gradient of 0-100% B over 50 min. After freeze-drying of the collected fraction, a white solid powder of peptide was obtained. Successful synthesis of KLA-Acm-R was confirmed with HPLC and mass spectrometry as shown in Figure S15A-B. c-FOS: m/z calculated for [M+2H]^2+^1684.94, found 1685.01, m/z calculated for [M+3H]^3+^ 1123.63, found 1123.68, m/z calculated for [M+4H]^4+^ 842.97, found 843.01.

### Immunoprecipitation and immunoblot analyses

For co-immunoprecipitation, His_6_- and EGFP-tagged proteins were overexpressed in MDA-MB-231 cells. Cells were lysed using ice-cold RIPA/PBS (33% v/v) containing an inhibitor cocktail (Roche Applied Science), sodium orthovanadate, and sodium fluoride. Total cell protein (1 mg) was incubated with 25 μl washed Protein G-magnetic beads (New England Biolabs) at 4°C for 1 h. Cleared lysates were incubated overnight at 4°C with 5 μg mouse monoclonal His_6_, mouse monoclonal GFP (Roche) or normal mouse IgG antibody, followed by 25 μl washed Protein G-magnetic beads at 4°C for 1 h. The immunoprecipitation matrix-antibody complex was washed three times with ice-cold RIPA/PBS (33% v/v), and bound proteins resolved via SDS-PAGE and subjected to immunoblot analysis. Signals were detected using enhanced chemiluminescence (Pierce). For endogenous co-immunoprecipitation, cells were lysed using ice-cold RIPA/PBS (33% v/v) containing an inhibitor cocktail (Roche Applied Science), sodium orthovanadate, and sodium fluoride. Rabbit monoclonal c-FOS, rabbit monoclonal c-JUN, normal mouse IgG and normal rabbit IgG antibodies (5 μg) were used for these experiments. Co-immunoprecipitation and immunoblot analyses were performed as described above. Uncropped images of the blots are shown in Figure S16.

### ChIP assay

ChIP assays were performed using the ChIP Assay Kit (Sigma-Aldrich) according to the manufacturer's protocol. Briefly, cancer cells incubated in a T25-flask were crosslinked by treatment with formaldehyde (final concentration, 1%) for 10 min at room temperature. After washing with PBS, cells were pelleted and resuspended in SDS lysis buffer (1% SDS, 10 mM EDTA, 50 mM Tris-HCI (pH 8.1), 1 mM DTT, and 1 mM PMSF). The lysates were then subjected to sonication to reduce the DNA length to between 500 and 1000 bp, diluted with dilution buffer (0.01% SDS, 1.1% Triton X-100, 1.2 mM EDTA, 16.7 mM Tris-HCI (pH 8.1), 167 mM NaCl), and pre-cleared by incubating with a Salmon Sperm DNA/protein A agarose-50% slurry for 60 min at 41C. The supernatant was incubated with anti-c-Fos (#2250S, Cell Signaling Technology) or anti-Rabbit normal IgG (#3900, Cell Signaling Technology) at 4°C overnight. Immunocomplexes were collected with the Salmon Sperm DNA/protein A agarose-50% slurry and eluted after extensive washings, and crosslinking was reversed by heating at 65°C, followed by treatment with 40 mg/ml proteinase K at 45°C for 60 min. DNA was recovered by phenol-chloroform/ ethanol precipitation, and was used as a template for PCR to amplify the target sites in the *CKS1B* promoter with the following primer pairs: PCR was performed with the following primer pairs: 5'-TTA GCC AAT GGC AGC GCG AGA TC-3' (forward) and 5'-TCC TTA TTG GAG GGA GTT CTC-3' (reverse). The PCR products were electrophoresed on a 1.5% agarose gel and stained with ethidium bromide (EtBr) (Sigma-Aldrich).

### HR and NHEJ assays

HR and NHEJ reporter assays were performed as previously described 54. The HR reporter assay system, pGCGFP (plasmid #31266), and the NHEJ reporter assay system, pimEJ5GFP (plasmid #44026), were purchased from Addgene. In the HR and NHEJ reporter assay systems, I-SceI endonuclease induces DSBs in a reporter plasmid that can only be repaired through HR and NHEJ, and successful repair is detected by monitoring EGFP fluorescence 54. For HR repair and NHEJ analyses, cells were transfected with pCBASceI (plasmid #26477, Addgene), selected with 1 mg ml^-1^ G418 for one week, and maintained in DMEM or RPMI-1640 containing 0.5 mg ml^-1^ G418. NQO1-expressing RKO (RKO/pshCont) and MDA-MB-231 (MDA-MB-231/pNQO1) cells were transfected with siCont or sic-Fos and NQO1-deficient RKO (RKO/pshNQO1) and MDA-MB-231 (MDA-MB-231/pCont) cells transfected with pCont or pc-FOS. After a 24 h incubation period, NQO1-expressing cells were transfected with pCont or pCKS1B and NQO1-deficient cells were transfected with siCont or siCKS1B. After further 24 h incubation, cells were transfected with pGCGFP (plasmid #31266, Addgene) or pimEJ5GFP (plasmid #44026, Addgene). Two days after transfection, cells were subjected to flow cytometry using the BD FACSMelody^TM^ instrument combined with a cell sorter (BD Biosciences). Three independent transfections were performed for each case.

### Ni-NTA-based pulldown assays

His_6_- and EGFP-tagged proteins were expressed in MDA-MB-231 cells and RKO cells, respectively. His_6_-proteins were affinity-purified and subsequently conjugated to Ni-NTA resin (Qiagen). His_6_-conjugated resin was incubated for 2 h at 4^o^C with 1 mg total protein of RKO cells. The resin was extensively washed, eluted, and subjected to immunoblot analysis.

### Small interfering RNA transfection

*NQO1*, *CKS1B* and *c-Fos* were subjected to RNA interference using a 19 bp (including a 2-deoxynucleotide overhang) small interfering RNA (siRNA). siRNAs against *NQO1* (CCGUACACAGAUACCUUGAdTdT), *CKS1B* (GUGACUUGCGGAUUUAUGUdTdT) and *c-Fos* (GUAUCUAGUGCAGCUGAUUdTdT) were purchased from Bioneer Corporation (Daejeon, Republic of Korea), with Stealth^TM^ RNAi (Invitrogen) used as a negative control. Cells were seeded in 25 cm^2^ flasks, grown to ~80% confluence, and transfected with siRNA duplexes using LipofectAMINE 2000 (Invitrogen) according to the manufacturer's protocol. After 48 h, cells were processed for analysis as indicated.

### Quantification of clonogenic death

Varying numbers of cells were plated on 6-well plates and irradiated (4 Gy) or left untreated. Cells were incubated for 14 days at 37^o^C and 5% CO_2_ to promote colony formation. The culture medium was decanted, and colonies were fixed with 95% methanol and stained with 0.5% crystal violet. The numbers of colonies (>50 cells) from triplicate dishes or plates were counted and the mean number of colonies formed by irradiated cells compared with that formed by untreated cells.

### Irradiation

Cells were exposed to X-rays using an X-RAD Ir160 X-ray irradiator (Accela)

### Immunofluorescence and confocal microscopy

Coverslip-mounted cells were fixed with 3.7% paraformaldehyde (PFA) and incubated in blocking solution (3% BSA) for 1 h at room temperature, after which they were incubated overnight at 4ºC with anti-phospho histone H2AX (ser139) (1:50; #05-636, EMD Millipore Corp), anti-c-Fos (1:50; #2250S, Cell Signaling Technology) or anti-NQO1 (1:25; 39-3700, Invitrogen) antibodies. Cells were washed three times with blocking solution and incubated with Alexa Fluor 488- or Alexa Fluor 594-conjugated secondary antibodies for 1 h. After washing twice with PBS, cells were stained with DAPI (Invitrogen) for 10 min to stain nuclei. Coverslips were washed three times with PBS and mounted onto slides using mounting reagent (Invitrogen), followed by analysis using laser-scanning confocal microscopy (TE2000-E; Nikon, Tokyo, Japan).

### Synchronization and cell cycle analysis

Cells were synchronized at the G1/S boundary using the double-thymidine block method. Cells were initially treated with 2 mM thymidine for 18 h, incubated in fresh medium without thymidine for 8 h, and retreated with thymidine for a further 16 h. Cells were finally released from arrest by replacing thymidine solution with fresh medium. For the G2/M boundary synchronization, cells were treated with 100 nM nocodazole for 22 h and released from the block by incubation with fresh medium. For analysis of CDK1 kinase activity and expression of cyclins and CDK, immunoblot analysis and CDK1 kinase assays were performed as described above. For cell cycle analysis, cells were fixed in cold 70% (vol/vol) ethanol, washed with cold phosphate-buffered saline (PBS) and stained with 40 μg/ml propidium iodide in the presence of 50 μg/ml ribonuclease A for 30 min at room temperature. Cellular DNA (10 000 cells per sample) was analyzed via flow cytometry (BD Biosciences).

### Immunohistochemistry

Human colon cancer and human breast cancer tissues were obtained from US Biomax (Rockville, MD). Paraffin-embedded sections were deparaffinized and rehydrated. Immunohistochemical analysis of NQO1 and CKS1 was performed with a Vectastain Elite ABC kit (Vector Laboratories Inc., Burlingame, CA) in keeping with the manufacturer's protocol. For antigen retrieval, sections were placed in citrate buffer (pH 6.0) and heated in a microwave oven for 10 min. For immunoperoxidase labeling, endogenous peroxidase was blocked with 0.3% H_2_O_2_ in absolute methanol for 15 min at room temperature. Sections were incubated overnight at 4°C with anti-NQO1 (1:100; NBP1-31355, Novus Biologicals) or anti-CKS1 (1:50; #36-6800, Invitrogen) antibody and washed with PBS containing 0.05% Trion X-100. Incubation with a secondary antibody and the peroxidase-antiperoxidase (PAP) complex was carried out for 30 min at room temperature. Immunoreactive sites were visualized using 3,3-diaminobenzidine tetrachloride (3,3′-DAB). Slices were subsequently counterstained with hematoxylin.

### Statistical analysis

All grouped data are presented as mean ± SD. Differences between groups were analyzed with ANOVA or Student's t-test using GraphPad Prism software. For survival analysis, Kaplan-Meier curves were generated using Prism software and log-rank analysis performed. All experiments were conducted at least in duplicate with three technical replicates.

## Supplementary Material

Supplementary figures and table.
