# Supplementary material for: NQO1 regulates cell cycle progression at the G2/M phase
Source: Theranostics. 2023 Jan 10;13(3):873–95. doi: 10.7150/thno.77444 (PMC9925316; doi:10.7150/thno.77444)

NQO1 regulates cell cycle progression at the G2/M phase

Oh et al.,

Supplementary Table 1 and Figures

Supplementary Table 1. Status of NRF2 and KEAP1 in the cell lines used in this study or causes of activation of Nrf2.

Cell line	Cancer type	Status of NRF2 and KEAP1 or causes of activation of Nrf2	Reference
**A549**	Lung cancer	KEAP1 G333C (homo)<br>KEAP1 promoter methylation	A
**RKO**	Colon cancer	Wild type Nrf2 (low basal level)<br>Wild type KEAP1	B
**MDA-MB-231**	Breast cancer	Wild type Nrf2 (low basal level)<br>Wild type KEAP1	C
**U87-MG**	Glioblastoma	WASP-interacting protein (WIP)<br>PTEN in the wild type of EGFR	D<br>E
**PC3**	Prostate cancer	Wild type KEAP1<br>GRP/Bip translocation	F<br>G
**MIA PaCa-2**	Pancreatic cancer	Wild type Nrf2 (low basal level)<br>Wild type KEAP1 (high level)	H

References for Supplementary Table 1

A. Singh A, Misra V, Thimmulappa RK, Lee H, Ames S, Hoque MO, et al. Dysfunctional KEAP1-NRF2 interaction in non-small-cell lung cancer. PLoS Med. 2006; 3, e420.

B. Sayin VI, LeBoeuf SE, Singh SX, Davidson SM, Biancur D, Guzelhan BS, et al. Activation of the NRF2 antioxidant program generates an imbalance in central carbon metabolism in cancer. eLife. 2017; 6, e28083.

C. Probst BL, McCauley L, Trevino I, Wigley WC, Ferguson DA. Cancer cell growth is differentially affected by constitutive activation of NRF2 by KEAP1 deletion and pharmacological activation of NRF2 by the synthetic triterpenoid, RTA 405. PLoS One. 2015; 10, e0135257.

D. Escoll M, Diego Lastra D, Robledinos-Antón N, Wandosell F, Antón IM, Cuadrado A. WIP modulates oxidative stress through NRF2/KEAP1 in glioblastoma cells. Antioxidants. 2020; 9, 773.

E. Luo S, Lei K, Xiang D, Ye K. NQO1 is regulated by PTEN in glioblastoma, mediating cell proliferation and oxidative stress. Oxid Med Cell Longev. 2018; 2018, 9146528.

F. Zhang P, Singh A, Yegnasubramanian S, Esopi D, Kombairaju P, Bodas M, et al. Loss of Keap1 function in prostate cancer cells causes chemo- and radio- resistance and promotes tumor growth. Mol Cancer Ther. 2010; 9, 336.

G. Bellezza I, Scarpelli P, Pizzo SV, Grottelli S, Costanzi E, Minelli A. ROS-independent Nrf2 activation in prostate cancer. Oncotarget. 2017; 8, 67506-18.

H. Lister A, Nedjadi T, Kitteringham NR, Campbell F, Costello E, Lloyd B, et al. Nrf2 is overexpressed in pancreatic cancer: implicantions for cell proliferation and therapy. Mol Cancer. 2011; 10, 37.

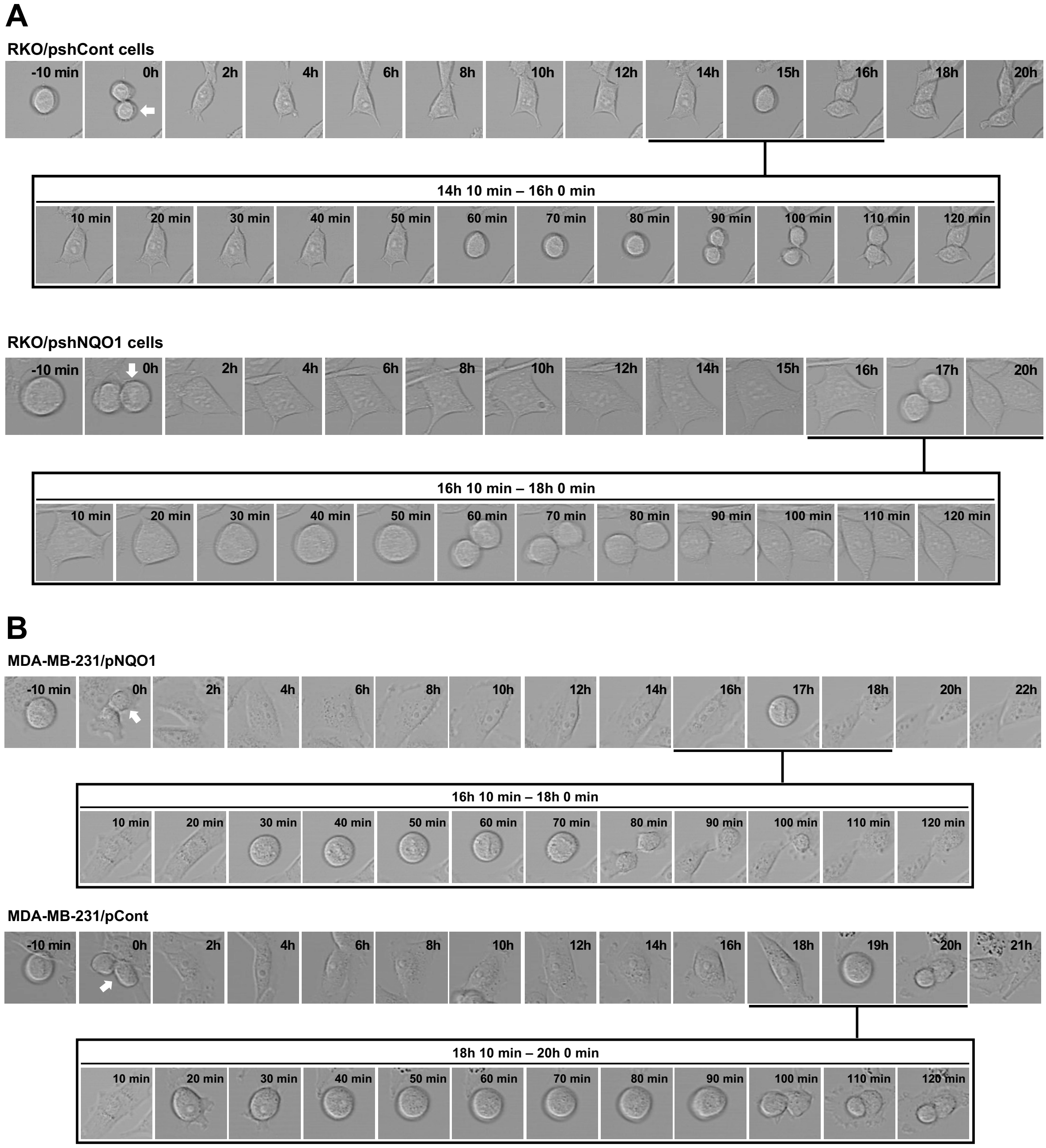

Figure S1. NQO1 regulates cancer cell proliferation. (A-B) RKO/pshCont and RKO/pshNQO1 cells **(A)** and MDA-MB-231/pNQO1 and MDA-MB-231/pCont cells **(B)** were seeded in 8-well chamber slides and cell cycle progression determined via confocal microscopy as indicated. White arrows signify the cells monitored for analysis of cell cycle progression.

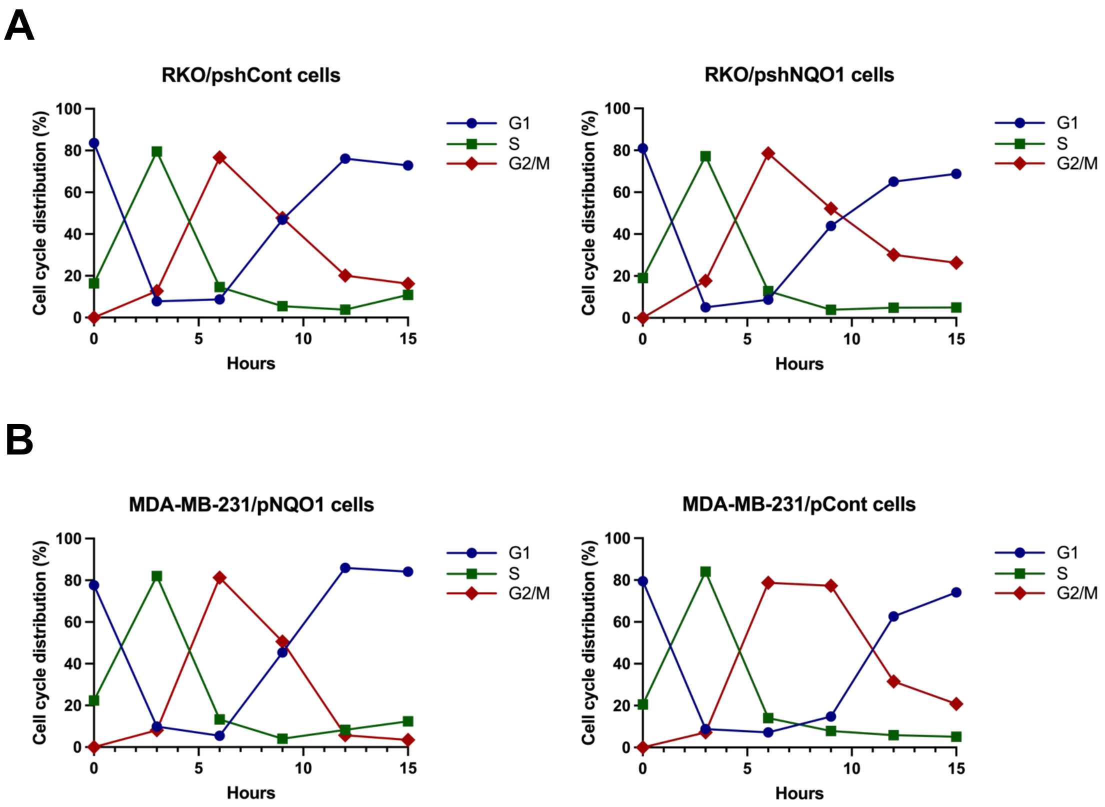

Figure S2. Quantification of cell cycle analysis. (A) Quantification of cell cycle analysis of RKO/pshCont cells and RKO/pshNQO1 cells in the flow cytometry analysis results of Figure 2A. **(B)** Quantification of cell cycle analysis of MDA-MB-231/pNQO1 cells and MDA-MB-231/pCont cells in the flow cytometry analysis results of Figure 2B.

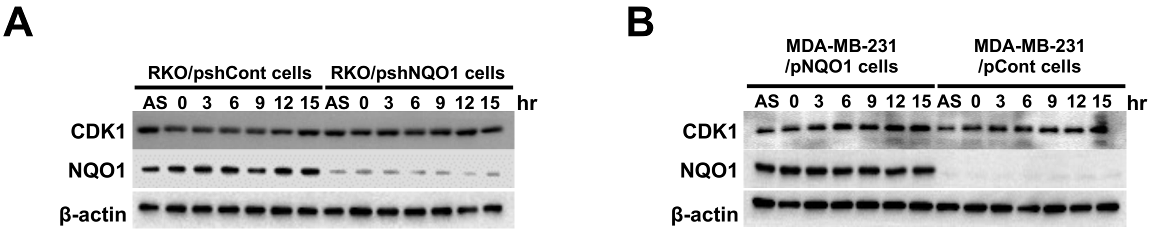

Figure S3. Effect of NQO1 on CDK1 expression in cancer cells. (A-B) RKO/pshCont and RKO/pshNQO1 cells **(A)** and MDA-MB-231/pNQO1 and MDA-MB-231/pCont cells **(B)** were synchronized at the G1/S boundary via double-thymidine blocking, released from synchronization, and harvested at the indicated times. Whole-cell lysates were analyzed by immunoblotting for CDK1, NQO1, and β-actin. AS indicates asynchronization.

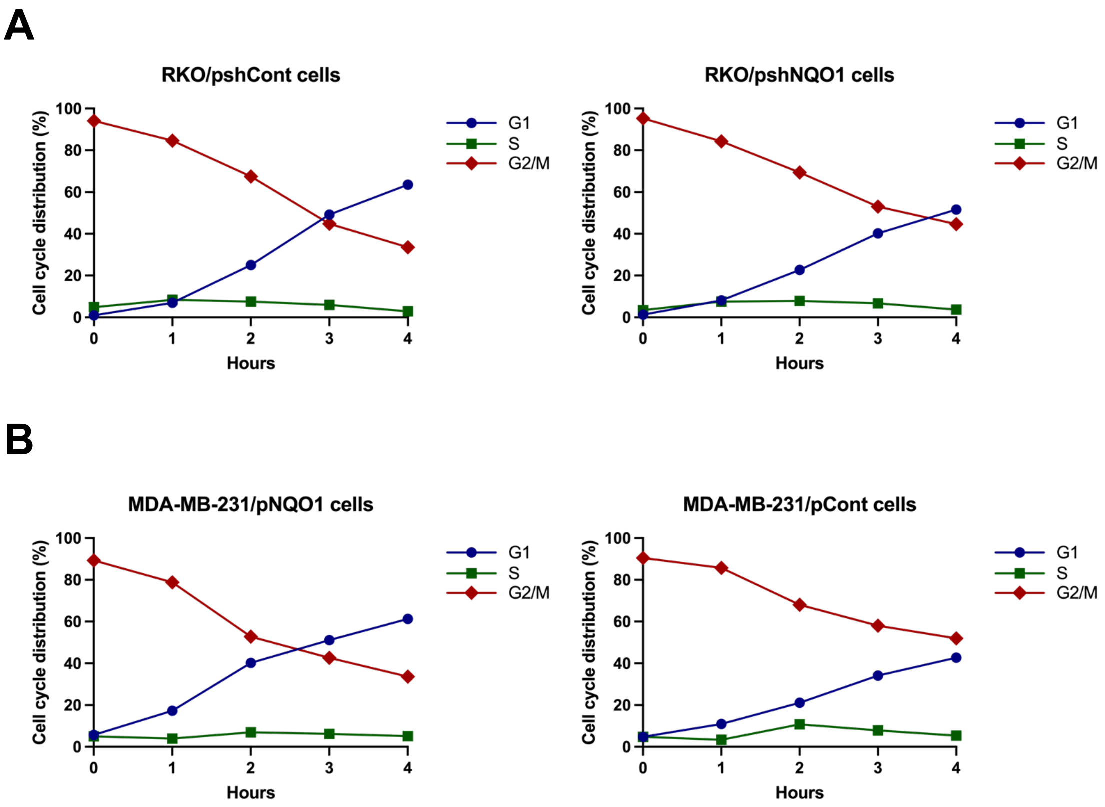

Figure S4. Quantification of cell cycle analysis. (A) Quantification of cell cycle analysis of RKO/pshCont cells and RKO/pshNQO1 cells in the flow cytometry analysis results of Figure 2G. **(B)** Quantification of cell cycle analysis of MDA-MB-231/pNQO1 cells and MDA-MB-231/pCont cells in the flow cytometry analysis results of Figure 2H.

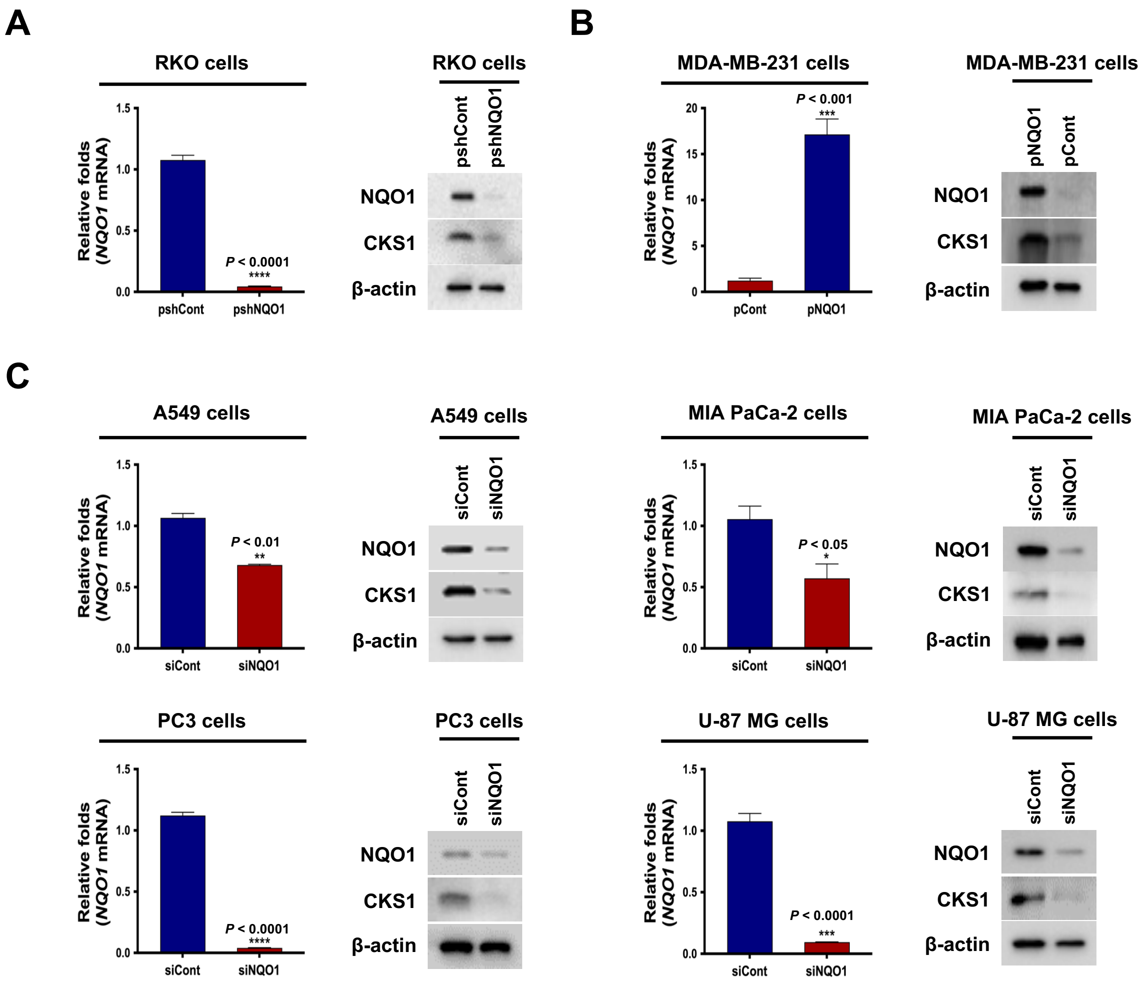

Figure S5. Effect of NQO1 on CKS1 expression in cancer cells. (A-B) The relative mRNA and protein expression levels of NQO1 and the relative protein expression levels of CKS1 in RKO/pshCont and RKO/pshNQO1 cells **(A)** and MDA-MB-231/pNQO1 and MDA-MB-231/pCont cells **(B)**. *NQO1* expression was examined via qPCR using *18S rRNA* as the internal control. All data are presented as mean ± SEM. *** *P* < 0.001 with unpaired *t*-test, **** *P* < 0.0001 with unpaired *t*-test. Protein expression was examined via immunoblotting. Whole-cell lysates were analyzed by immunoblotting for NQO1, CKS1, and β-actin. **(C)** The relative mRNA and protein expression levels of NQO1 and the relative protein expression levels of CKS1 in A549, MIA-PaCa-2, PC3, and U87-MG cells transfected with siCont or siNQO1. *NQO1* expression was examined via qPCR using *18S rRNA* as the internal control. All data are presented as mean ± SEM. * *P* < 0.05 with unpaired *t*-test, ** *P* < 0.01 with unpaired *t*-test, *** *P* < 0.001 with unpaired *t*-test A, **** *P* < 0.0001 with unpaired *t*-test. Protein expression was examined via immunoblotting. Whole-cell lysates were analyzed by immunoblotting for NQO1, CKS1, and β-actin.

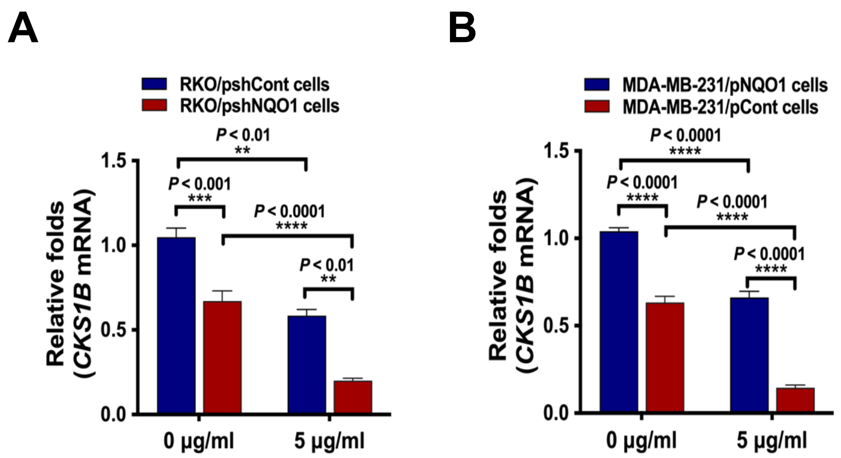

Figure S6. Effect of NQO1 on *CKS1B* mRNA stability in cancer cells. **(A-B)** RKO/pshCont and RKO/pshNQO1 cells **(A)** and MDA-MB-231/pNQO1 and MDA-MB-231/pCont cells **(B)** were incubated with 5 µg/ml actinomycin D for 2 h and harvested. *CKS1B* expression was examined via qPCR using *18S rRNA* as the internal control. All data are presented as mean ± SEM. ** P < 0.01 with ANOVA, *** P < 0.001 with ANOVA, **** P < 0.001 with ANOVA.

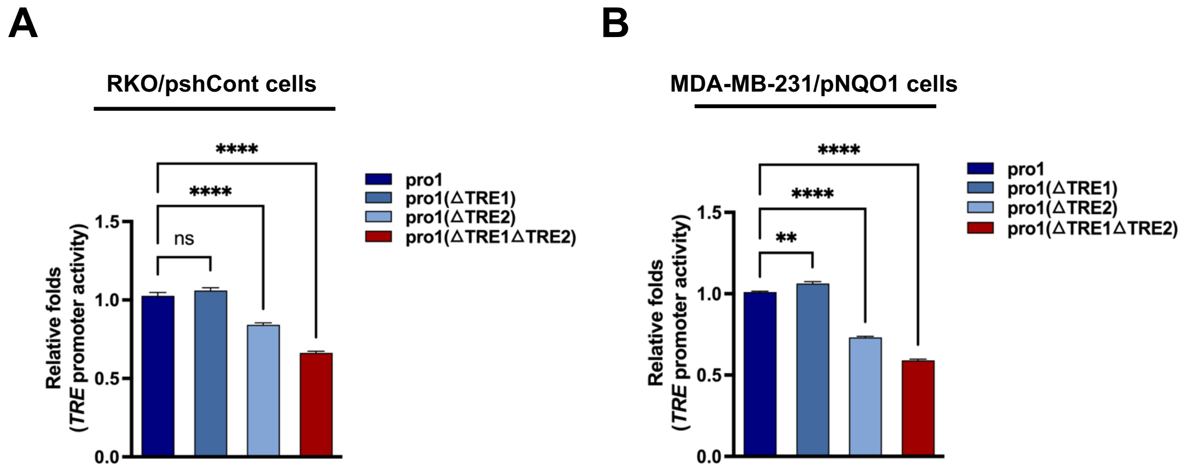

Figure S7. Effect of the TRE region of the *CKS1B* promoter on *CKS1B* expression in cancer cells. (A-B) RKO/pshCont cells **(A)** and MDA-MB-231/pNQO1 cells **(B)** were transfected with wild type pro1, TRE1 deletion mutant pro1, TRE2 deletion mutant pro1, or TRE1 and TRE2 deletion mutant pro1 and control pRL-*luc.* After 4 h, cells were washed with PBS and incubated with the appropriate medium for 48 h. Luciferase activity was normalized to that of *Renilla* (mean ± SEM). ** $P < 0.01$ with ANOVA. **** $P < 0.001$ with ANOVA. NS indicates no significance.

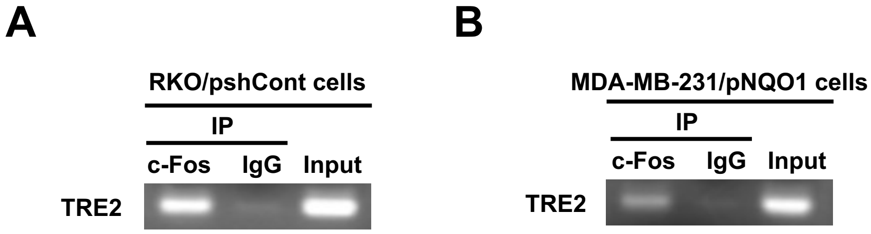

Figure S8. ChIP assay to verify the binding of c-Fos to TRE2 on CKS1B promoter. **(A, B)** RKO/pshCont cells **(A)** and MDA-MB-231 cells **(B)** were treated with formaldehyde for crosslinking, and DNA was extracted and immunoprecipitated with ant-c-Fos antibody and anti-normal IgG. After reversal of crosslinking, DNA was purified and used as a templated for PCR to amplify TRE2 site. The PCR products were electrophoresed on 1.5% agarose gel and visualized by staining with EtBr.

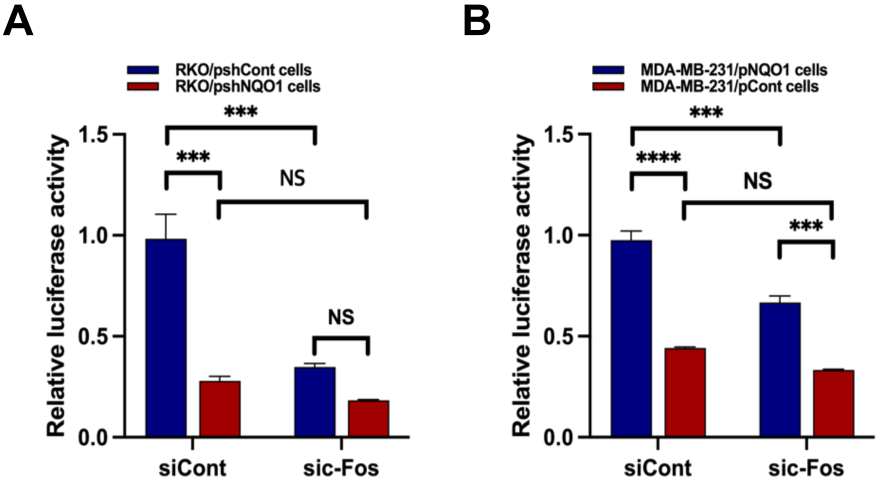

Figure S9. Effect of c-Fos on *CKS1B* expression in NQO1-expressing and NQO1-deficient cancer cells. (A-B) RKO/pshCont and RKO/pshNQO1 cells **(A)** and MDA-MB-231/pNQO1 and MDA-MB-231/pCont cells **(B)** were transfected with siCont or sic-Fos, incubated for 1 day, transfected with pTRE-*luc* and the transfection control pRL-*luc*. After 4 h, cells were washed with PBS and incubated with the appropriate medium for 48 h. Luciferase activity was normalized to that of *Renilla* (mean ± SEM). *** $P < 0.001$ with ANOVA. **** $P < 0.001$ with ANOVA. NS indicates no significance.

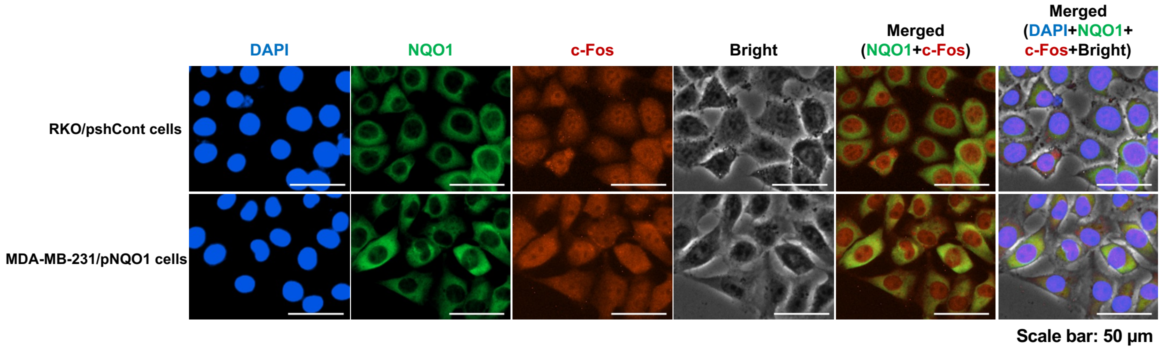

Figure S10. NQO1 and subcellular localization of c-Fos. Subcellular localization was visualized by confocal microscopy. RKO/pshCont cells and MDA-MB-231/pNQO1 cells were fixed for immunofluorescence analysis with the indicated antibodies. Nuclear staining is indicated by using DAPI.

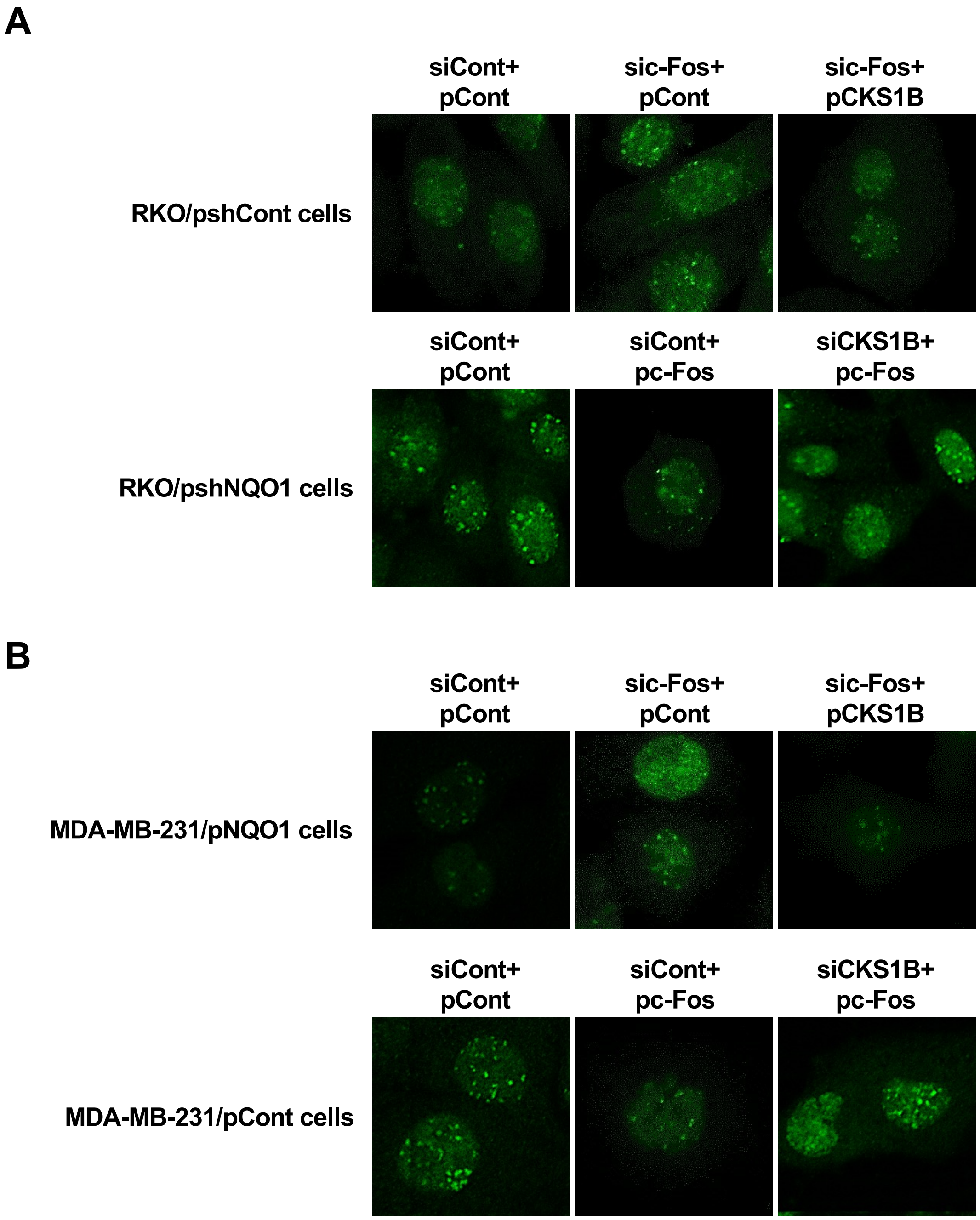

Figure S11. Representative figures of Fig. 6C and D. (A-B) RKO/pshCont cells **(A)** and MDA-MB-231/pNQO1 cells **(B)** were transfected with siCont and pCont, sic-Fos and pCont or sic-Fos and pCKS1B, and RKO/pshNQO1 cells **(A)** or MDA-MB-231/pCont cells **(B)** transfected with pCont and siCont, pc-Fos and siCont or pc-Fos and siCKS1B. After 48 h of incubation, various quantities of cells were plated on an eight-well chamber slide, cultured for 16 h, and irradiated with 0 or 4 Gy, followed by culture for 24 h. Cells were fixed with 3.7% PFA and immunofluorescence performed using anti-phospho histone H2AX.

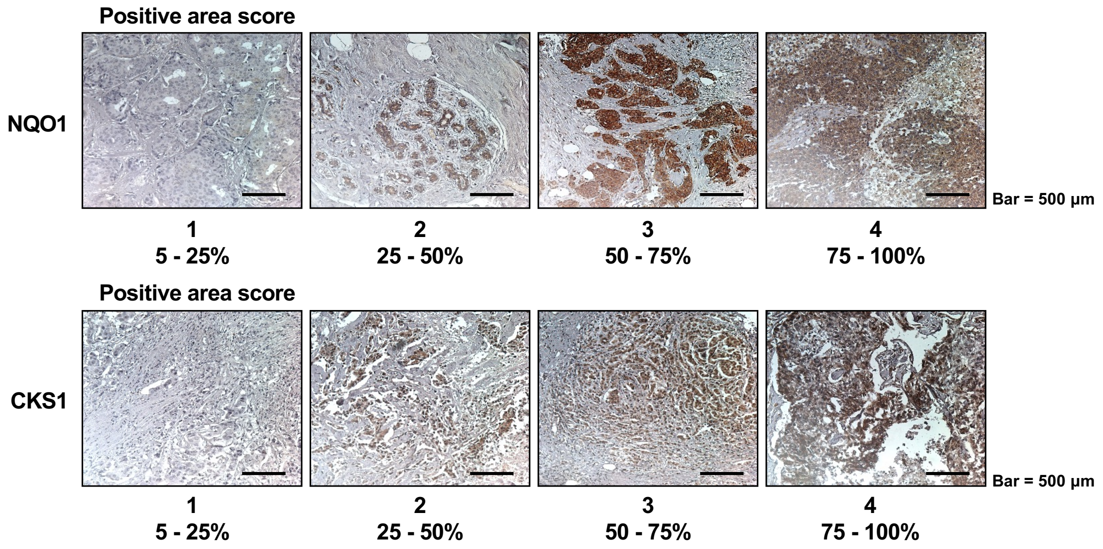

Figure S12. Histopathological scoring criteria used for analysis of NQO1 and CKS1 staining in human clinical tumor tissue. IHC scores of NQO1 and CKS1 were defined by positive area score (5-25%,1; >25-50%,2; >50-75%,3; >75-100%,4).

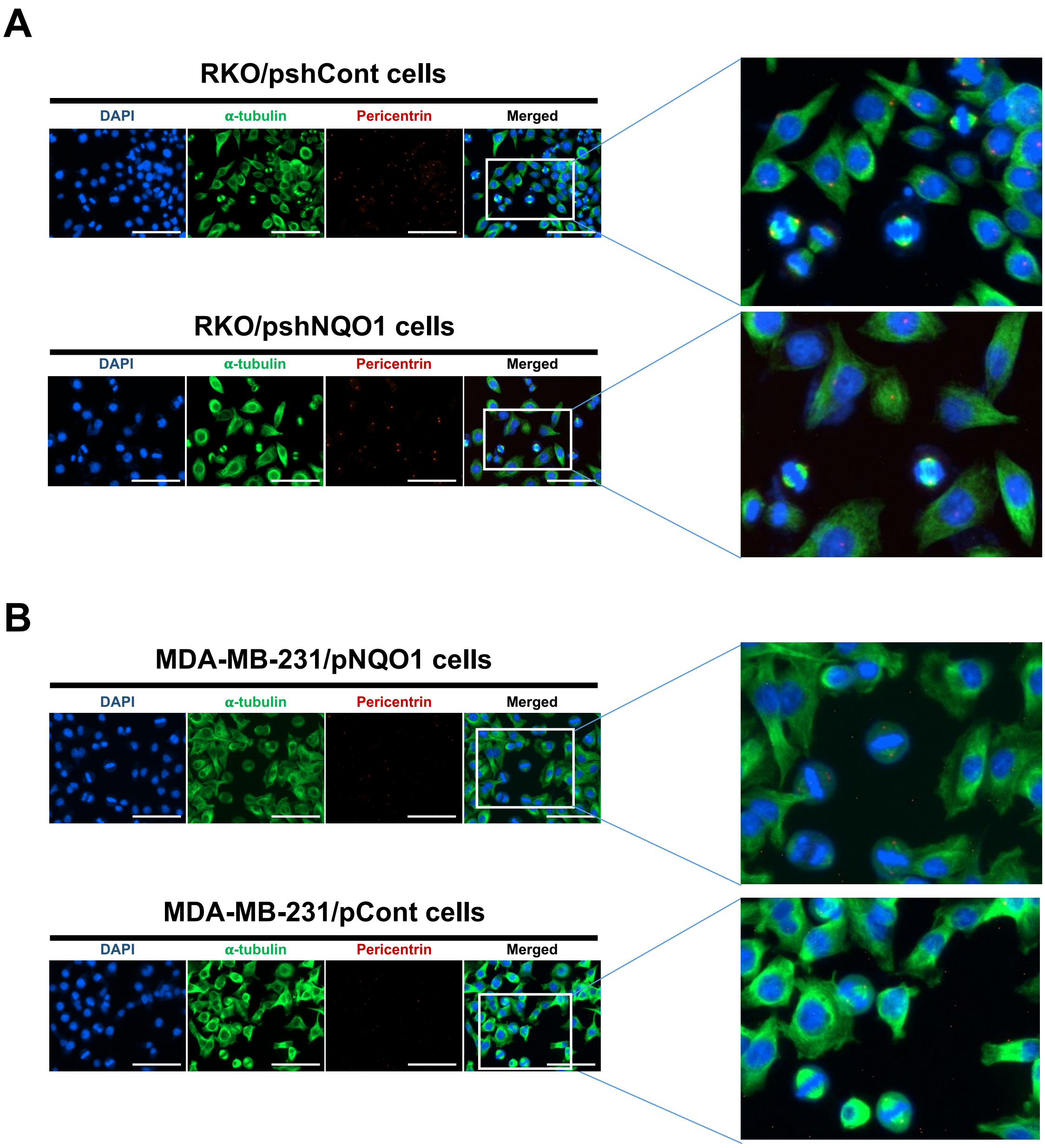

Figure S13. Effect of NQO1 on mitotic spindle formation in cancer cells. (A-B) RKO/pshCont and RKO/pshNQO1 cells **(A)** and MDA-MB-231/pNQO1 and MDA-MB-231/pCont cells **(B)** were fixed with 3.7% PFA and immunofluorescence performed using anti-α-tubulin and anti-pericentrin.

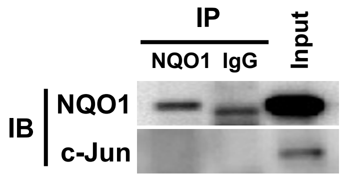

Figure S14. Analysis of interaction between NQO1 and c-Jun. Whole-cell extracts of RKO/pshCont cells were immunoprecipitated with anti-NQO1 and anti-IgG (negative control) and analyzed by immunoblotting with anti-NQO1 anti-c-Jun antibodies.

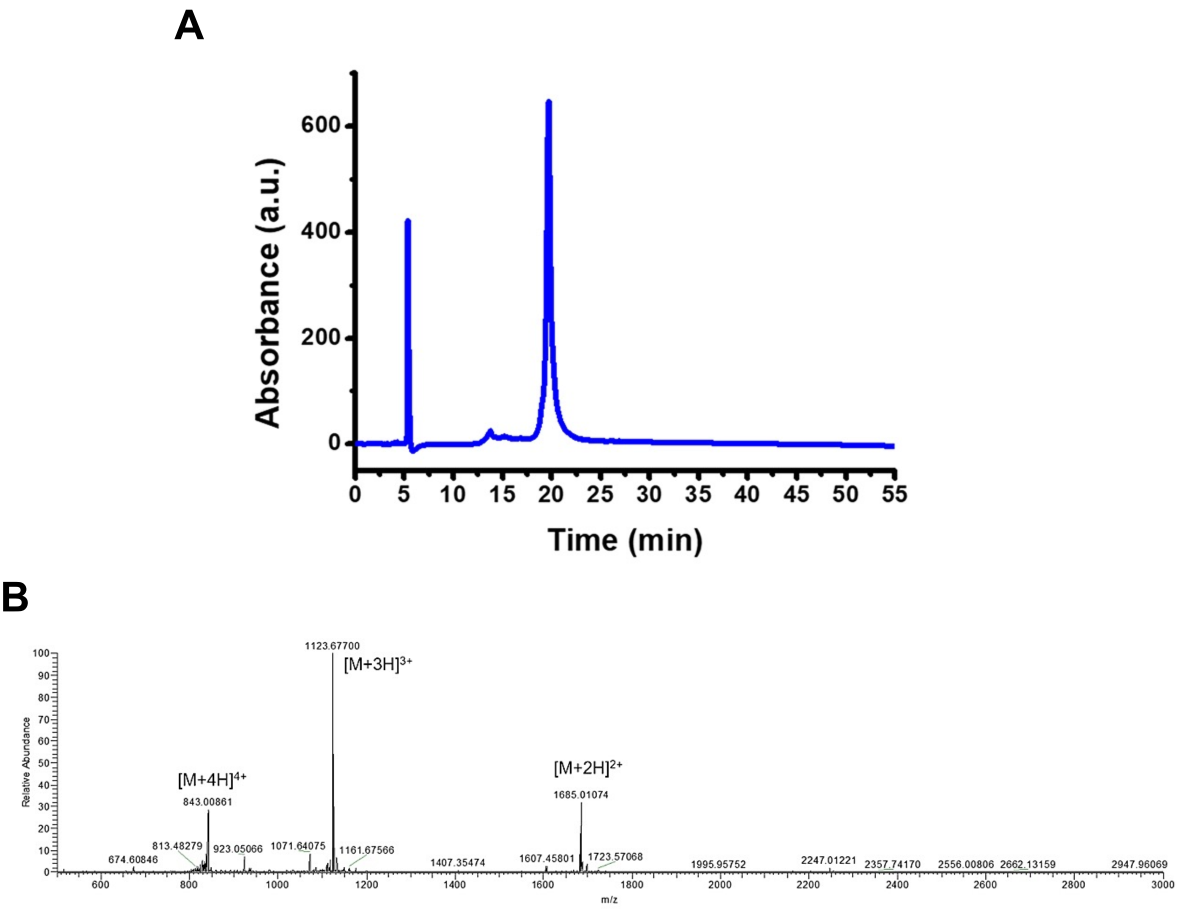

Figure S15. Analysis of synthesized c-Fos peptide. (A) HPLC chromatogram of c-Fos peptide**. (B)** LC-Mass spectrum of c-Fos peptide.

Figure S16.

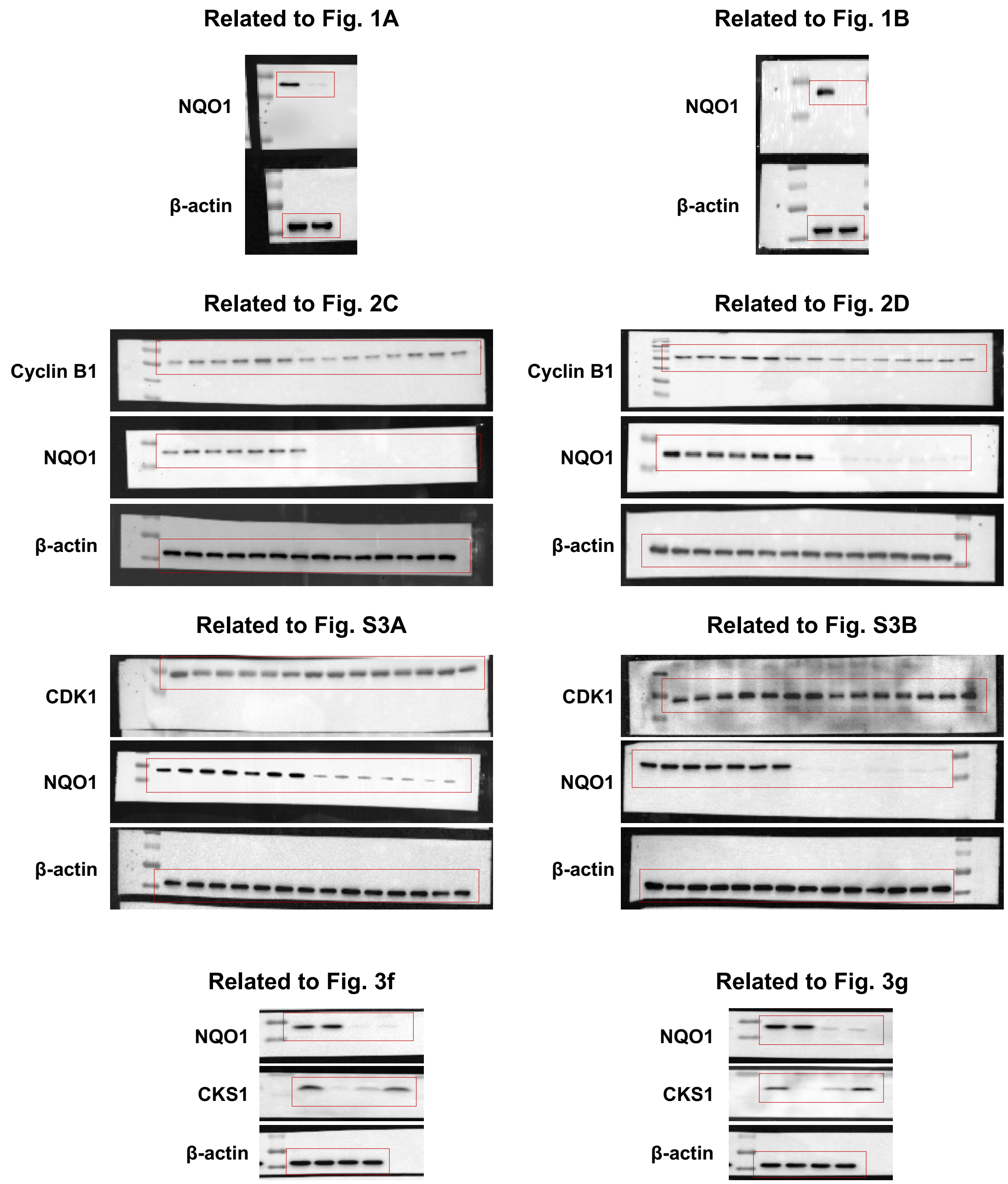
Related to Fig. 1A
NQO1
β-actin
Related to Fig. 1B
NQO1
β-actin
Related to Fig. 2C
Cyclin B1
NQO1
β-actin
Related to Fig. 2D
Cyclin B1
NQO1
β-actin
Related to Fig. S3A
CDK1
NQO1
β-actin
Related to Fig. S3B
CDK1
NQO1
β-actin
Related to Fig. 3f
NQO1
CKS1
β-actin
Related to Fig. 3g
NQO1
CKS1
β-actin

Related to Fig. S5A

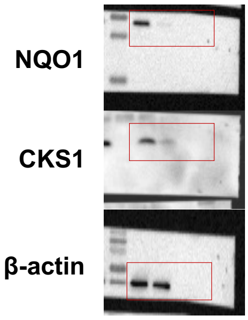

Related to Fig. S5B

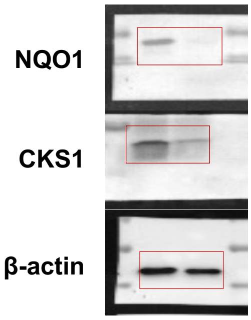

Related to Fig. S5C

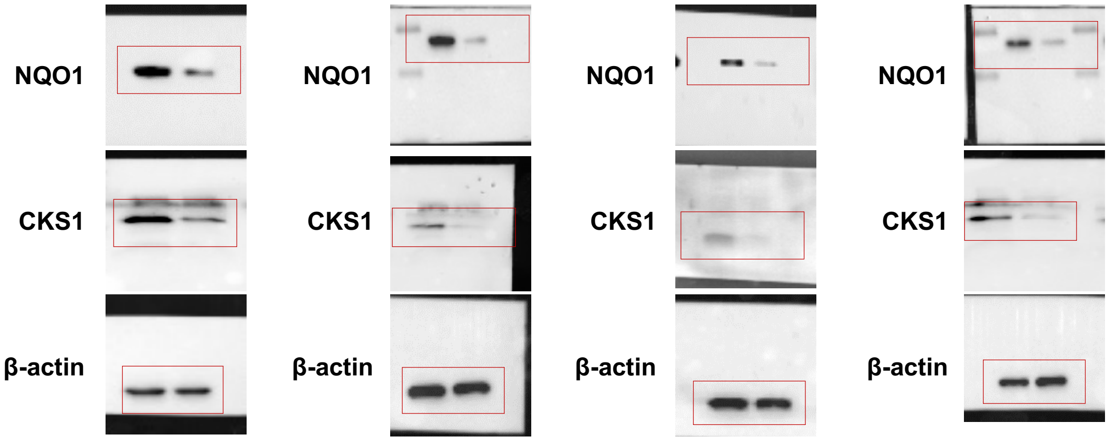

Related to Fig. 4G

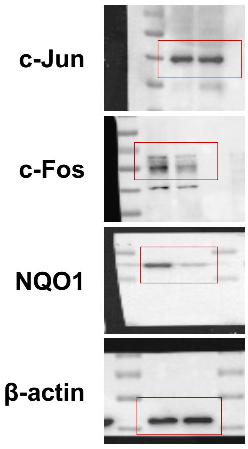

Related to Fig. 4H

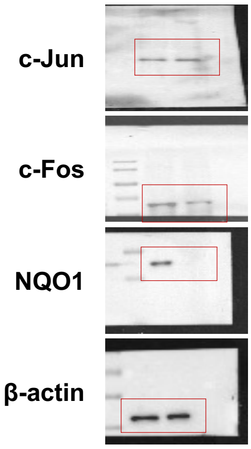

Related to Fig. 4I

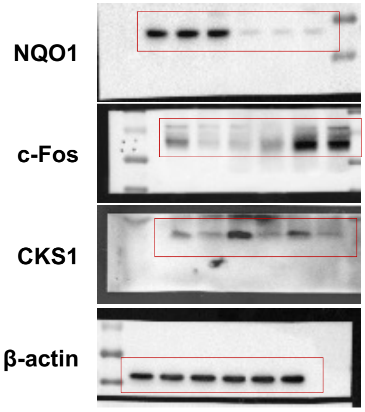

Related to Fig. 4J

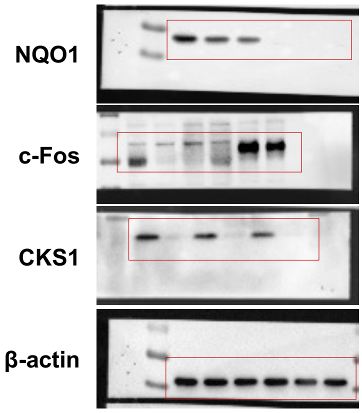

Related to Fig. 5A

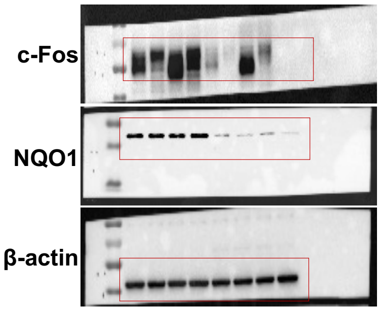

Related to Fig. 5B

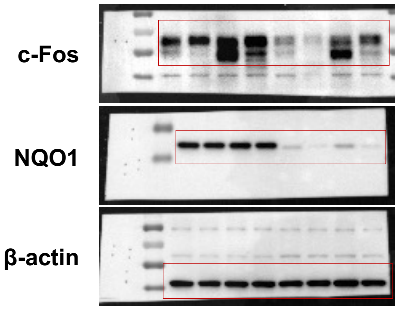

Related to Fig. 5C

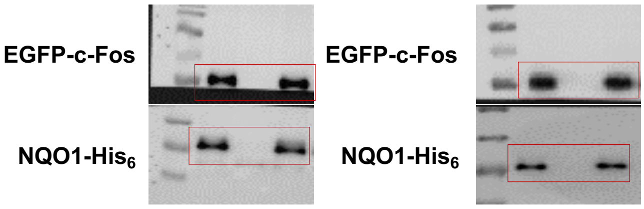

Related to Fig. 5D

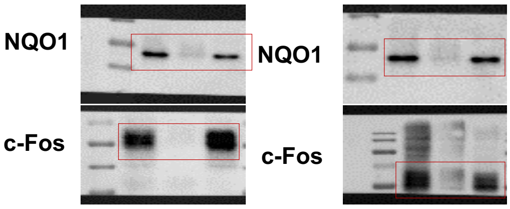

Related to Fig. 5E

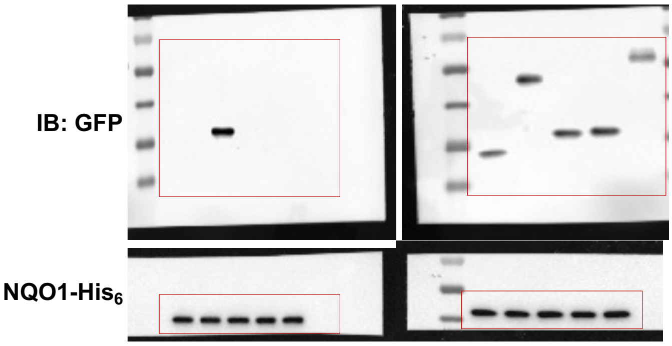

Related to Fig. 5F

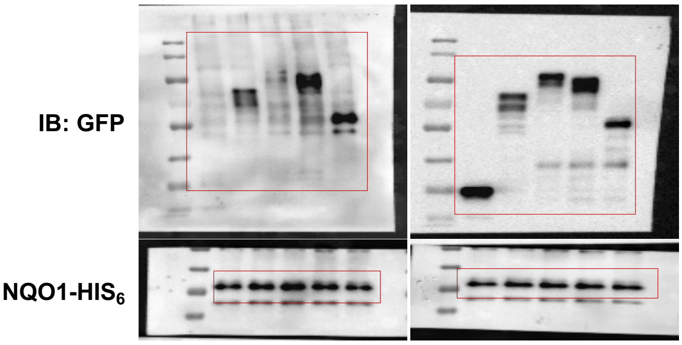

Related to Fig. 5H

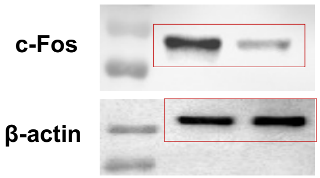

Related to Fig. S8

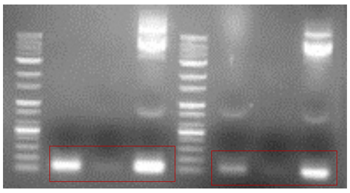

Related to Fig. S13

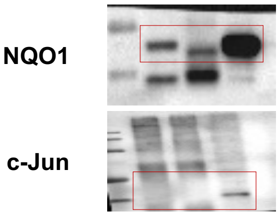

Supplement: Supplementary file 1 — Supplementary figures and table. [file thnov13p0873s1.pdf]
